# Leg-body coordination strategies for obstacle avoidance and narrow space navigation of multi-segmented, legged robots

**DOI:** 10.3389/fnbot.2023.1214248

**Published:** 2023-11-08

**Authors:** Nopparada Mingchinda, Vatsanai Jaiton, Binggwong Leung, Poramate Manoonpong

**Affiliations:** ^1^Bio-Inspired Robotics and Neural Engineering Laboratory, School of Information Science and Technology, Vidyasirimedhi Institute of Science and Technology, Rayong, Thailand; ^2^Embodied AI and Neurorobotics Laboratory, SDU Biorobotics, The Mærsk Mc-Kinney Møller Institute, University of Southern Denmark, Odense, Denmark

**Keywords:** bio-inspired robotics, millipede, recurrent neural network, single recurrent neurons, legged robot, neural dynamics, temporal delays, hysteresis

## Abstract

**Introduction:**

Millipedes can avoid obstacle while navigating complex environments with their multi-segmented body. Biological evidence indicates that when the millipede navigates around an obstacle, it first bends the anterior segments of its corresponding anterior segment of its body, and then gradually propagates this body bending mechanism from anterior to posterior segments. Simultaneously, the stride length between pairs of legs inside the bending curve decreases to coordinate the leg motions with the bending mechanism of the body segments. In robotics, coordination between multiple legs and body segments during turning for navigating in complex environments, e.g., narrow spaces, has not been fully realized in multi-segmented, multi-legged robots with more than six legs.

**Method:**

To generate the efficient obstacle avoidance turning behavior in a multi-segmented, multi-legged (millipede-like) robot, this study explored three possible strategies of leg and body coordination during turning: including the local leg and body coordination at the segment level in a manner similar to millipedes, global leg amplitude change in response to different turning directions (like insects), and the phase reversal of legs inside of turning curve during obstacle avoidance (typical engineering approach).

**Results:**

Using sensory inputs obtained from the antennae located at the robot head and recurrent neural control, different turning strategies were generated, with gradual body bending propagation from the anterior to posterior body segments.

**Discussion:**

We discovered differences in the performance of each turning strategy, which could guide the future control development of multi-segmented, legged robots.

## 1. Introduction

Legged locomotion control has been studied over decades. Previously studies mostly included biped (He et al., 2019; Akkawutvanich et al., 2020; Yao et al., 2022), quadruped (Chen et al., 2019; Fukui et al., 2019; Zhang et al., 2021), hexapod (Bai et al., 2019; Wang et al., 2020; Ouyang et al., 2021; Ma et al., 2022), and octopod (Grzelczyk et al., 2019; Miguel-Blanco and Manoonpong, 2020) robots. More recently, there has been an increased interest in robots with more legs and longer bodies. Studies involving millipede-inspired robots used various approaches to generate the locomotion of the robots, e.g., piezoelectric motors (Oldham et al., 2009; Hoffman and Wood, 2011; Avirovik and Priya, 2013; Avirovik et al., 2014), and magnetic fields (Venkiteswaran et al., 2019, 2020; Wang et al., 2022). On the other hand, other studies have used neural mechanisms to implement reactive and adaptive locomotion control of millipede-inspired robots (Kano et al., 2017; Miguel-Blanco and Manoonpong, 2020; Ambe et al., 2022; Mingchinda et al., 2022). While these studies evaluated the robot locomotion behaviors in various environments, the leg and body coordination of such robots for obstacle avoidance and narrow space navigation in complex environments remains unclear.

In contrast to artificial multi-legged systems, biological multi-legged systems (like millipedes) show impressive leg-body coordination. They, along with centipedes, are known as myriapods under the class of *Diplopoda*^1^ (Garcia et al., 2020). Previously, a notable example of a centipede-like robot over rugged terrain demonstrated the flexibility of multi-segmented and multi-legged robots, with potential applications in search and rescue, extraterrestrial exploration, etc. (Chong et al., 2023). However, it remains unclear how a millipede-like robot with features similar to centipedes can be effectively controlled in more complex environments, such as narrow spaces. One key feature of the millipedes is that their locomotion systems are both robust and adaptable to various terrains, which enables them to fulfill biological functions such as digging through decomposed matters (Spinello and Fattahi, 2017; Joly et al., 2020; Marek et al., 2021). As the millipede traverses through an environment, its body moves forward by the direct waves that propel the legs from posterior to anterior legs (Kuroda et al., 2014; Ambe and Aoi, 2019; Ambe et al., 2021, 2022). Further, the millipede also coordinates the strides of the legs during navigating through obstacles within the environment (Garcia et al., 2015). Millipedes possess a sophisticated neural system that enables them to utilize sensory information to achieve motor control and navigate through various environments (Francisco et al., 2015; Reboleira and Enghoff, 2018).

How could a creature with more than six legs (like millipede) coordinate the movements of numerous legs with long, segmented bodies for obstacle avoidance and complex environment navigation? A vital feature of its movement is its turning behavior for obstacle avoidance, which involves coordination between the turned body segments and legs. Specifically, during turning, the stride length is reduced between each pair of legs in the inner turning curve as the millipede turns. This stride length reduction enables the millipede to make a turn without collisions between its numerous legs (Barnwell, 1965; Hembree, 2009; Garcia et al., 2015). This important feature has been implemented in millipede inspired robots (Long et al., 2002; Aoi et al., 2016; Mingchinda et al., 2022; Shao et al., 2022).

However, previous studies have not investigated the underlying control mechanism and strategies that efficiently enable the millipede robots to perform a coordinated and smooth turning behavior, especially in complex environments such as narrow spaces. In contrast to the turning mechanisms in limbless robots like snakes (Wu and Ma, 2013), which leverage obstacles for body-body coordination, resulting in obstacle-aided locomotion, this study focuses on two key dimensions of coordination in millipedes: leg-body coordination and body-body coordination. Both dimensions require a control system that properly uses the sensory input information from the environment to produce the desired adaptive behavior of the robot in response to the obstacle. A previous attempt has been made to control the turning behavior of multi-segmented robots at the segment level by passively following the movement of the first segment (Aoi et al., 2016) and by actively controlling the body segments with temporal delays (Mingchinda et al., 2022). Nevertheless, both studies were limited in determining how robots can effectively coordinate the leg and body adaptations to deal with obstacles within the environment.

A smooth and coordinated turning behavior can be implemented by the cooperation between the body segments and legs via the use of the hysteresis effect (Pasemann, 1993a,b, 1997). The hysteresis effect can act as a short-term memory of a neural control system, where the maintenance of output signals can induce temporal delays between each neural activity. In robotics, the hysteresis effect has been utilized in neural-based control in legged robots to control locomotion and sensory response to environmental stimuli (Hülse et al., 2007; Manoonpong et al., 2008, 2010, 2012; Grinke et al., 2015; Mingchinda et al., 2022). In our previous study, the dynamics of single recurrent neurons (hysteresis effect) was exploited to generate turning behaviors with different turning angles depending on the weight of the recurrent neurons (Mingchinda et al., 2022). Further, single recurrent neurons can be connected in series with excitatory connections between each recurrent neuron to produce the gradual turning behavior seen in millipedes. This is because the first recurrent neuron in the series processes the changes in the sensory information from the environment before propagating them to the posterior neurons (Mingchinda et al., 2022). Coupled with the temporal delays from the hysteresis effect, a series of single recurrent neurons can be used to control the signals sent to each leg or body joint to generate the millipede-like turning behavior resulting from temporal delays. Therefore, the hysteresis effect is effective in manipulating the turning behavior of the millipede in accordance with the environmental changes.

We extend our previous research (Mingchinda et al., 2022) to explore different strategies for coordinating leg and body movement in response to obstacles in complex environments, i.e., narrow spaces. To build on the previous research, our work aims to implement a millipede-inspired leg-body coordination strategy during turning in narrow environments. This strategy will be compared to other two leg-body coordination strategies to elucidate the uniqueness of millipede-like turning behavior, and identify the potential applications of this leg-body coordination strategy in bio-inspired robotics. We established three leg-body coordination strategies:

- Local leg and body coordination at the segment level (LCS).
- Simultaneous leg amplitude reduction (SAR).
- Global leg phase reversion (GPR).

The first strategy (LCS) is based on the turning behavior of real millipedes (Garcia et al., 2015). Here, the stride length between each leg inside the turning curve of the millipede is reduced to facilitate turning at the segment level. However, in the other two strategies (SAR, GPR), all legs inside the turning curve change simultaneously in response to changes in the sensory inputs from the environment during turning. The SAR strategy is based on insect turning (Rosano and Webb, 2006), while the GPR strategy is a typical engineering approach (Manoonpong et al., 2007). To investigate their behaviors, we first investigated the turning behavior using each leg-body coordination strategy against a solid wall. Subsequently, the efficiency of each strategy in obstacle avoidance in narrow spaces in terms of speed and task completion time was tested.

## 2. Materials and methods

### 2.1. Millipede-inspired robot

**Figure 1:**
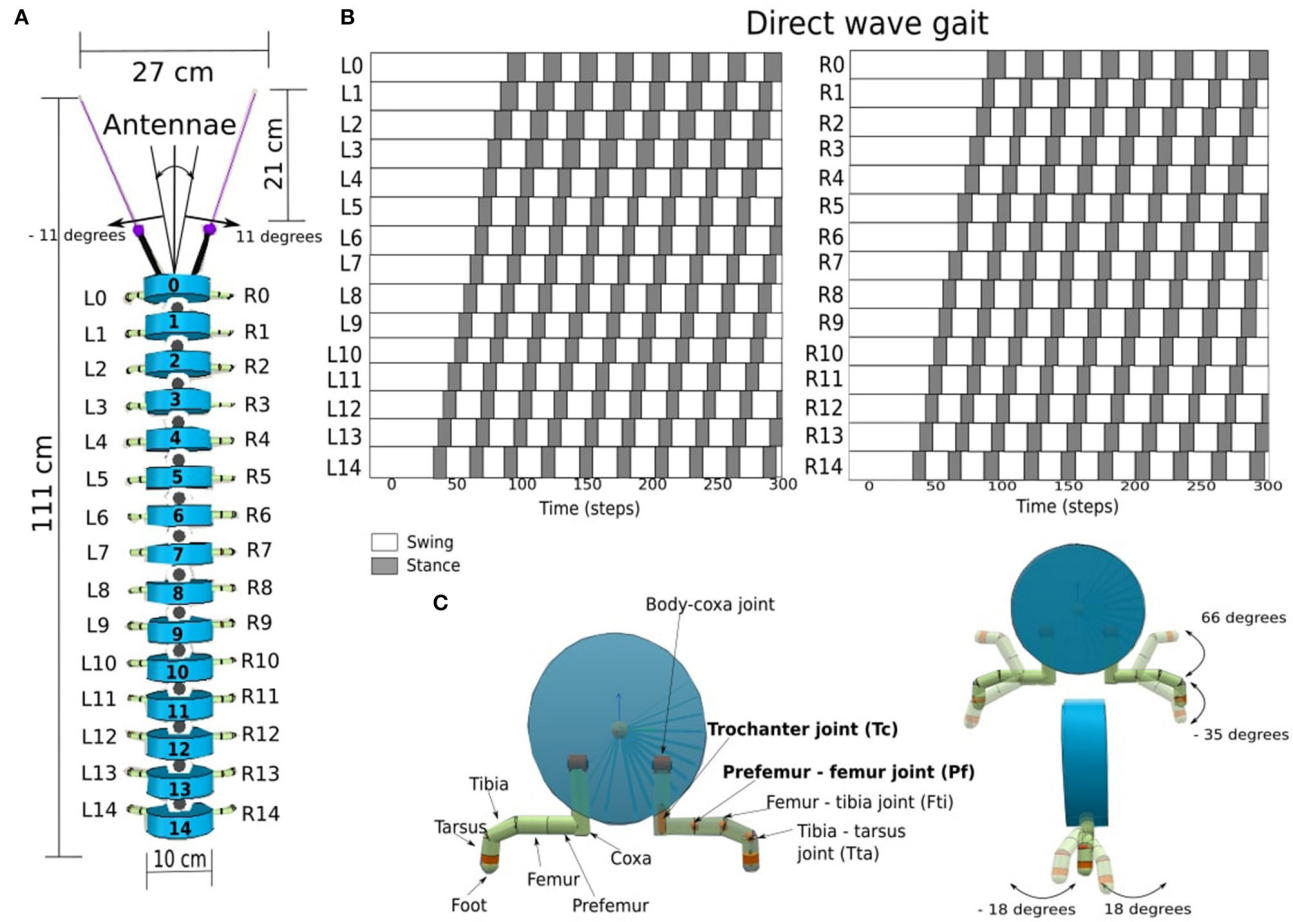
**(A)** The components and dimensions of the millipede robot, consisting of the antennae, the legs and the segmented body. The first segment is numbered 0, and the last segment is numbered 14. **(B)** The direct-wave gait pattern of the millipede robot for the left legs (*L*_0_ – *L*_14_), and the right legs (*R*_0_ – *R*_14_), with dark squares representing the stance (legs on the ground), and the white squares representing the swing (legs off the ground). **(C)** The cross-section of a body segment, showing the leg components and their associated joints. In each leg, the actuated joints are the trochanter joint (*Tc*) and the prefemur-femur joint (*Pf*), which are hereby denoted in bold, while other joints are fixed at certain positions.

We used Coppelia Sims version 4.4.0 (Rohmer et al., 2013) to simulate the millipede robot to investigate different leg-body coordination strategies (LCS, SAR, GPR). The millipede robot is a simplified model of real-world millipedes *A. virginiensis* (Garcia et al., 2015), consisting of fifteen segments and one pair of legs per segment. This resulted in a total of 30 legs (Figure 1A). Our millipede robot has short legs in comparison to its body length, which is different from centipedes, where the legs are longer in relation to the body length. The parameters of our robot model, such as body diameter size (10 cm) and leg length (7.4 cm), were derived and scaled based on a millipede model proposed by Enghoff (1992). The robot driven by the neural control walked with a direct-wave gait, where waves propagate from posterior to anterior legs as seen in real millipedes (Figure 1B). Each leg consists of six separate segments that are connected by revolute joints. However, for simplicity, only the trochanter (*Tc*) and prefemur-femur (*Pf*) joints are actuated (Figure 1C in bold).

There is one revolute joint between each body segment for left or right movement. There are 14 revolute body joints with an angle range of −11 to 11 degrees from left to right to prevent collision between each body segment. At the head of the body, which corresponds to the segment 0 (Seg 0), we attached the antennae containing infrared sensors (IR) at the tips. The sensor range of both IR sensors is 21 cm. Each IR sensor feedback is mapped to the range between (−1, ..., 1), where −1 corresponds to “no obstacle detected” and values above −1 up until 1 correspond to “obstacle detected”. The other values between −1.0 and 1.0 signify obstacles detectable by IR sensors within the range of 21 to 0 cm.

At each leg, the trochanter joint (*Tc*) controls the movement forward (+) and backward (−), while the prefemur-femur joint (*Pf*) controls the movement upward (+) and downward (−). The *Tc* and *Pf* joints are revolute joints, with angle ranges of −18 to 18 degrees and −35 to 66 degrees, respectively (Figure 1C). Together with the segment, the length between the left and right legs is 15 cm. The total length of the robot is 111 cm, including the length of all the segments, antennae, and IR sensors at the head.

### 2.2. Millipede-inspired neural control system

**Figure 2:**
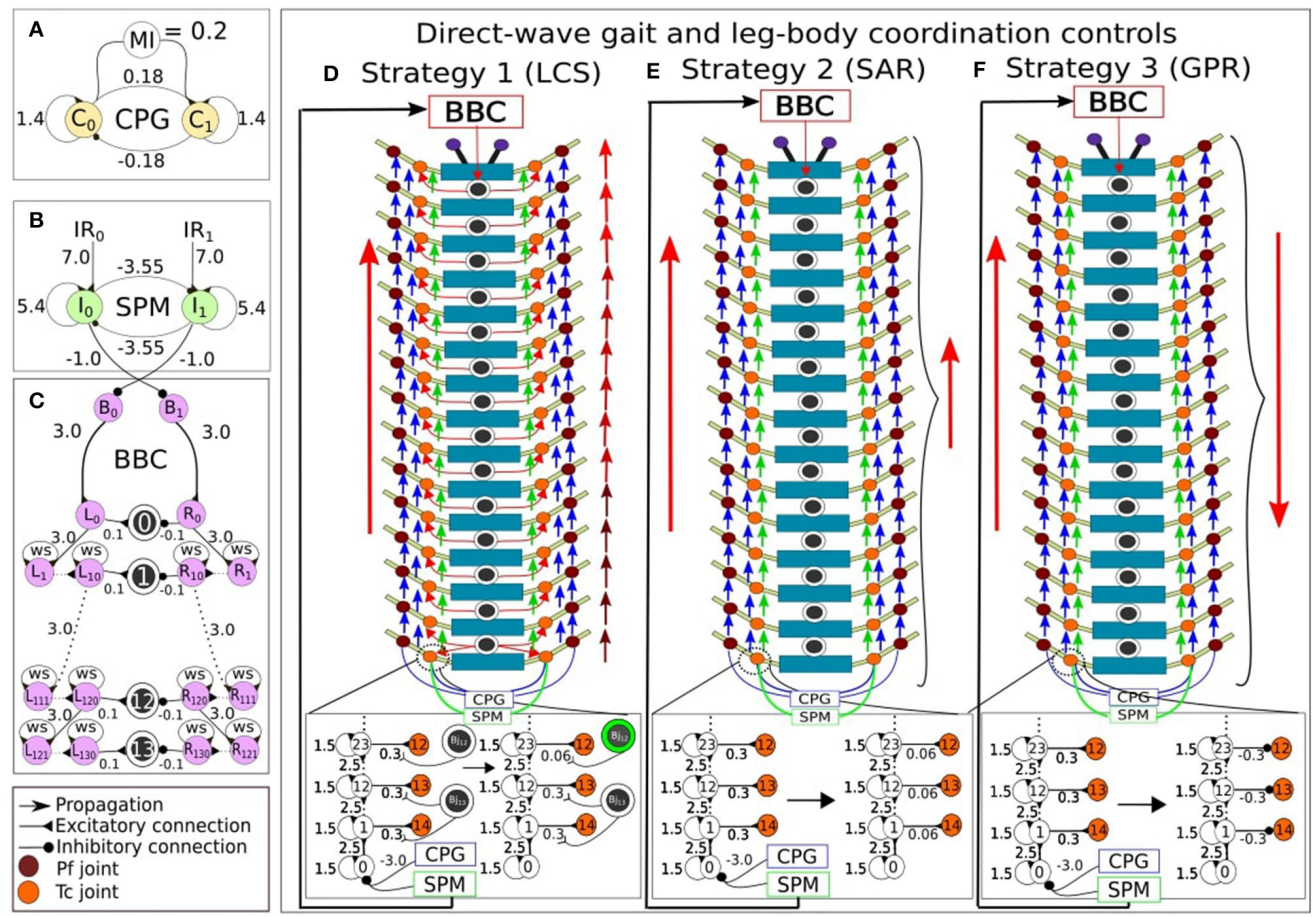
The modular neural control networks controlling the leg and body adaptation of the millipede robot. **(A)** The central pattern generator (CPG) produces sinusoidal waves that enable movement in each actuated joint. **(B)** The sensory processing module (SPM) processes incoming sensory inputs from the antennae, and sends outputs to adapt the movement in each actuated joint. **(C)** The body bending control (BBC) module contains two series of single recurrent neurons that produce temporal delays. For the direct-wave gait and leg-body coordination controls, the CPG and SPM project their outputs to *Tc* recurrent series, and the former to *Pf* recurrent series. After, every eleventh recurrent neurons from *Tc* and *Pf* series connect to their respective *Tc* and *Pf* joint motor neurons in order to achieve walking behavior like direct-wave gait. Additionally, three leg-body coordination strategies are implemented by modulating the input weight of *Tc* motor neurons to generate adaptive turning behavior. **(D)** LCS: If an obstacle is on the left, the amplitude between each pair of right legs becomes reduced, starting from the first two pairs of legs due to actions to the body joints mediated by the BBC. This is indicated by the gradually changing color gradient of the right red arrows (in case of a right turn) indicating the direct-wave gait, showing the movement of first pairs of legs in lighter red color to represent amplitude reduction. Also, a green circle indicates an activated Bj, which leads to the reduction in the connection weight between the recurrent neuron and *Tc* joint in that segment. **(E)** SAR: In contrast, the amplitudes between all pairs of legs inside the turning curve are reduced simultaneously, as indicated by the smaller red arrow on the right side in case of a right turn. **(F)** GPR: The direction of the leg movement inside the turning curve is reversed. Note that the red arrows indicate leg adaptation strategies. Blue arrows indicate the flow of CPG output signals from one joint to the next, and green arrows indicate the flow of SPM output signals from one joint to the next.

We utilized neural control for controlling the movement and turning behavior of the robot, based on similar studies on multi-legged robots (Aoi et al., 2017; Yasui et al., 2017; Homchanthanakul and Manoonpong, 2021). Consistent with our previous study (Mingchinda et al., 2022), the neural control system (Figure 2) consists of a central pattern generator module (CPG, Figure 2A), a sensory processing module (SPM, Figure 2B), and a body bending control module (BBC, Figure 2C) for leg-leg coordination control (i.e., direct-wave gait control, see Section 2.2.1 and Supplementary material), body-body coordination control (see Section 2.2.2 and Supplementary material), and leg-body coordination control (see Section 2.3 and Supplementary material). The CPG generates periodic oscillatory signals that drive the motors of the *Tc* and *Pf* joints via direct-wave gait control (Figure 1C), enabling the rhythmic pattern of leg movements. Here, there is a modulatory input (*MI*) of 0.2 for normal stepping frequency. Along with the CPG, the SPM processes the sensory information received from the IR sensors. The two recurrent neurons *I*_0_ and *I*_1_ at the SPM acts as a low-pass filter for the noisy sensory signals (Figure 2B). Moreover, the inhibitory connections between neurons *I*_0_ and *I*_1_ of the SPM reduce the interference between information received from the left and right IR sensors when both antennae detect an obstacle by inhibiting the activities of the opposing neurons. The *I*_0_ and *I*_1_ outputs are projected to adapt the movements of the left and right *Tc* joints as well as the body joints for obstacle avoidance. All neurons inside the CPG, SPM, and BBC modules are modeled using discrete-time non-spiking neurons. The activity of each neuron is defined as:


(1) $$\begin{array}{l} a_{i}\left(t\right)=\overset{n}{\underset{j=1}{\sum }}W_{ij}o_{j}\left(t-1\right)+B_{i},i=1,...,n, \end{array}$$

where *n* refers to the number of units. *B*_*i*_ is a fixed bias term of the neuron *i*. *W*_*ij*_ denotes the synaptic connection from neurons *j* to *i*. *o*_*i*_ denotes the output of neuron *i*, which is calculated using a hyperbolic tangent (*tanh*) activation function (*o*_*i*_ = *tanh*(*a*_*i*_) ∈ [−1, 1]). The setup of the neural model and structure leads to direct-wave gait control (interlimb coordination or coordination between legs) and body bending control (body-body coordination or coordination between body segments), each of which is further described below. Our current study focuses mainly on the leg-body coordination control strategies for efficient turning behavior of the millipede robot. Therefore, we shortly describe here the concept and basic setup of the neural control system for direct-wave gait control and body bending control, and detailed information on the CPG, SPM and BBC modules can be found in Mingchinda et al. (2022) and Supplementary material.

#### 2.2.1. Leg-leg coordination control

**Figure 3:**
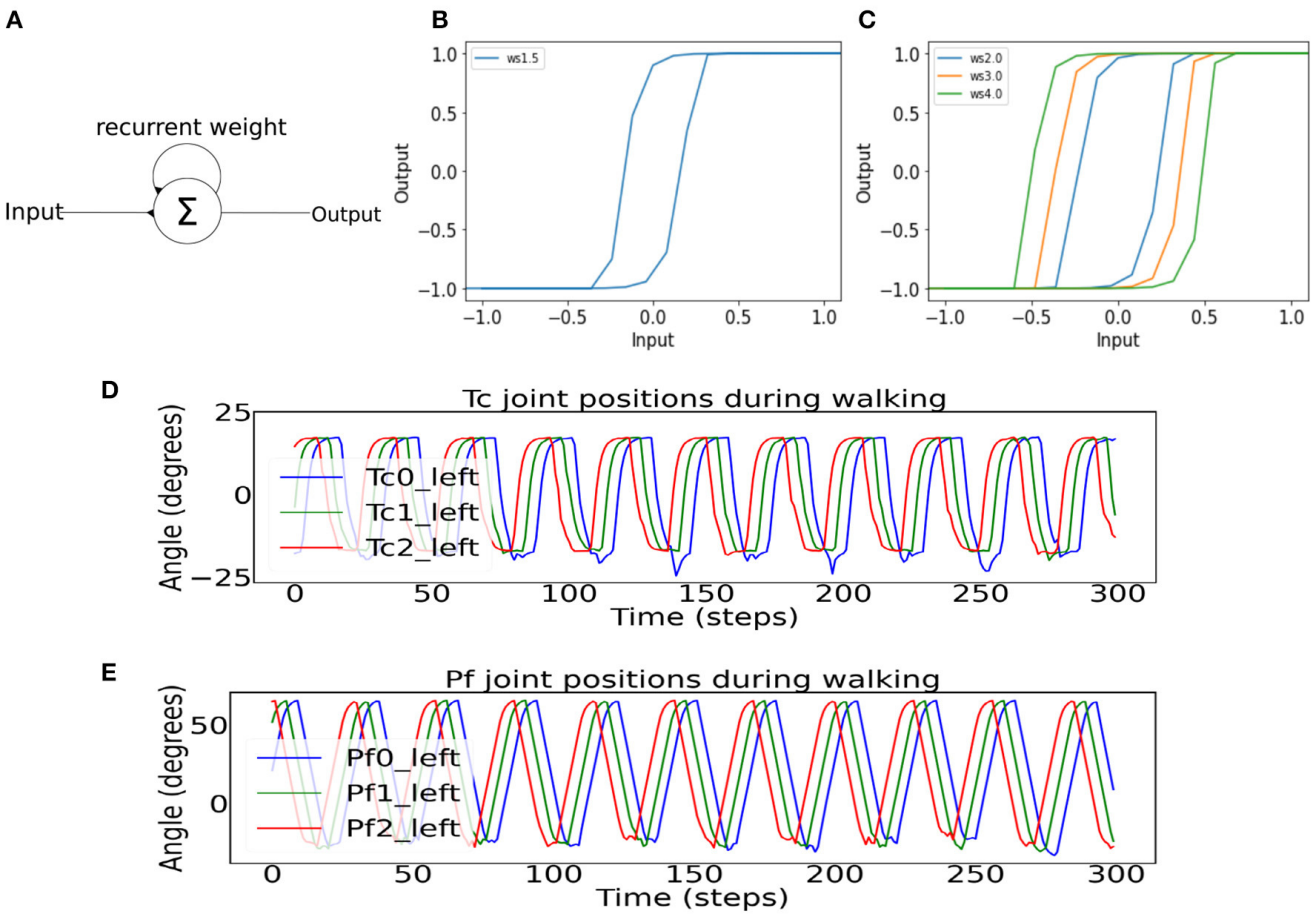
**(A)** A single recurrent neuron containing two excitatory connections, with one being self-connection and the other one being the input connection. **(B)** The hysteresis effect of recurrent neurons in the single recurrent neuron series controlling the phases of the *Tc* and *Pf* joints. The recurrent connection weight is 1.5 to control the stride length. **(C)** The hysteresis effect shows different hysteresis loop sizes for different self-connection weights used in the BBC. **(D)** Example of the *Tc* joint positions of the first three left legs show direct-wave gait propagating from the posterior to anterior legs. Between the time period of 100 and 250 steps, there are some noises in the troughs of the waves as a result of the robot's up and down motion during locomotion. **(E)** Example of the *Pf* joint positions of the first three left legs shows direct-wave gait propagating from the posterior to anterior legs during straight walking.

To implement the leg-leg coordination for generating a direct-wave gait, the neurodynamics of single recurrent neurons was employed, which produced the hysteresis effect and acted as a low-pass filter for the sensory inputs from the environment (Manoonpong et al., 2010) (Figure 3A). Therefore, the hysteresis effect essentially filters unwanted sensory noises in the environment. Further, with more single neurons connected in the series (Figure 2C), multiple hysteresis effects are produced, which lead to temporal delay. The further a recurrent neuron along the series, the higher the temporal delay. These differences in the size of temporal delays of each recurrent neuron in the series can be used to control the time step in which each *Tc* and *Pf* joint become activated. The further the single recurrent neuron from the sensory input, and the larger the self-excitatory weight (Figures 3B, C), the larger the hysteresis loop, which results in the longer temporal delay. However, the phases of the first left and right legs will eventually coincide with those of the first legs of the next wave period (Figure 1B), resulting in a wave gait pattern due to the overlapping of periodic leg joint activities. When the outputs of the first single recurrent neurons in the series are projected to the posterior legs before the anterior legs, the wave gait seen in millipedes can be reproduced in the robot. Also, the frequency of the wave pattern can be manipulated by changing the value of the *MI*, with lower *MI* leading to lower wave frequency as shown in Homchanthanakul and Manoonpong (2021).

In this study, we empirically adjusted the number of neurons in the left and right of *Tc* and *Pf* recurrent series controlling the left and right legs to achieve the direct-wave gait. As a result, each series contains a total of 155 recurrent neurons, which are connected to each other via excitatory connections with a weight at each connection of 2.5. For the connection between the recurrent neuron series and the *Tc* and *Pf* joints, the second rather than the first single recurrent neuron was projected to the first posterior leg pair to mitigate the effect of potential differences in the signal pattern, as the first single recurrent neuron in the series receives inputs directly from the SPM (Figure 2). After the first posterior leg pair, each subsequent pair receives input from every eleventh neuron in the left or right single recurrent neuron series. The *Tc* joints, starting from the last pair of legs, received inputs from every eleventh single recurrent neuron (Figure 2). As for the *Pf* joints, the left and right single recurrent neuron series governing the *Pf* joints received inputs from the CPG, with every single recurrent neuron connected by excitatory connections with a weight of 2.5 (Figure 2). The discrepancy between the outputs to the *Tc* and *Pf* resulting from temporal differences in the signal delays lead to the activation of *Tc* before *Pf*, thereby forming in the desired direct-wave gait that resembles real millipedes. Specifically, the legs are lifted (*Pf* action) before stepping forward (*Tc* action). The network parameters, including the recurrent neuron weight and connection weight between each neuron, are determined by testing the behavior of the leg joints at weights ranging from 1.0 to 3.0 based on our previous study (Mingchinda et al., 2022). For example, a new weight is chosen if the wave gait is not produced. Note that the equations for the neural activations of the single recurrent neuron series and motor neurons mentioned above can be found in Supplementary material.

#### 2.2.2. Body-body coordination control

Under the same principles of hysteresis and low-pass filter, the body-body coordination control [here described as body bending control (BBC, Figure 2C)] was designed based on the evidence that the millipede navigates gradually from an obstacle segment-by-segment (Garcia et al., 2015). Previous studies (Pasemann, 1993a) on neural dynamics showed that different excitatory recurrent weight (*ws*) values produced various sizes of the hysteresis (Figure 3C), which provided a useful tool for manipulating the turning angle of the millipede. Specifically, our previous study has also shown that higher recurent weights (*ws*) produced higher turning angles in a millipede-like robot when faced with a wall directly in front of it. Another important factor during turning in narrow spaces is the robot's ability to maintain its bent position, which is achievable using the hysteresis effect as the output signal is maintained despite diminishing input signals (Mingchinda et al., 2022). In other words, the hysteresis effect creates a short-term memory in the system, enabling the millipede robot to remain bent and navigate away from an obstacle to avoid re-collision. Note that the equations for the neural activations of the body joints, containing the recurrent weight term *ws*, can be found in Supplementary material.

Although we can now control the body segments for turning during obstacle avoidance and control the legs for performing the wave gait (Figures 3D, E), a problem still remains. The problem is to determine the coordination between leg and body movements such that the turning behavior of the millipede robot enables effective navigation in narrow environments. Contrary to our previous study, whereby we relied on the SPM inputs to control the motion of both the body and leg joints, here we attempt to address this question by investigating three different body-leg coordination strategies.

### 2.3. Exploring different leg-body coordination strategies

During turning, millipedes coordinate their leg and body segments to perform smooth motion (Garcia et al., 2015). This is achieved by reducing the stride length between the leg pairs inside the turning arc during turning. Previously, we relied solely on the sensory inputs from the SPM to coordinate the leg and body movements (Mingchinda et al., 2022). This previous approach is comparable to GPR (Figure 2F). If changes in both leg and body joints are according to the sensory inputs, the leg and body joints can automatically adjust to the changes in sensory information in the environment by reversing the phases of leg joints and rotating body joints away from obstacles. This results in a behavior where all the leg joints change their behavior regardless of the changes in the body segment angle in a similar manner to other animals using tripod-based gaits (Strauss and Heisenberg, 1990; Szczecinski et al., 2018; Nirody et al., 2021). However, this assumption is opposite to what is observed in millipedes in the real world, where changes in the leg and body joints are more coordinated at the local (i.e., segment) level. If only the first two body segments bend, there will be no changes in the stride lengths between other leg pairs apart from the first two to allow the millipede to turn its head.

**Figure 4:**
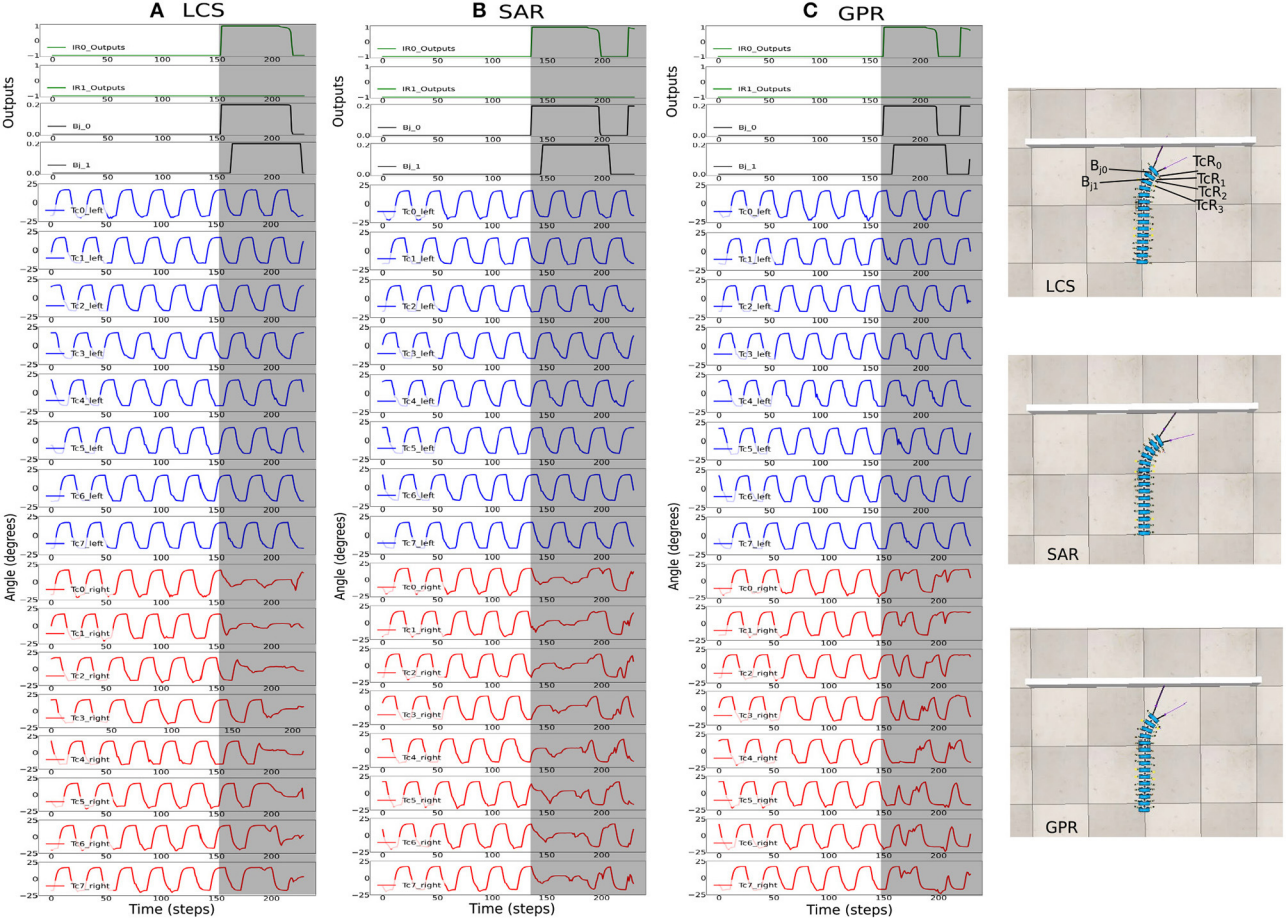
The three turning strategies, with graphs showing body joint outputs and changes in the angles of the *Tc* joints for right legs during a right turn. **(A)** Strategy 1 (local leg and body coordination at the segment level, LCS). In this case, the stride length of the legs inside the turning arc, i.e., right legs in this case, is reduced if the body joint between the two leg pairs surrounding it bends as a result of the detection of an obstacle. **(B)** Strategy 2 [simultaneous leg amplitude reduction (SAR)]. In this case, the stride length between all leg pairs inside the turning arc are simultaneously reduced as the robot makes a turn. **(C)** Strategy 3 [global leg phase reversal (GPR)]. Here, the movement direction of the legs inside the turning arc switches direction as the robot turns, resulting in the reversal of the phases of those legs.

To test the significance of the leg and body coordination at the local level, or the segment level, we produced three different leg and body coordination strategies for our millipede robot during turning (Figure 4). These strategies are:

- Local leg and body coordination at the segment level (LCS),
- Simultaneous leg amplitude reduction (SAR),
- Global leg phase reversion (GPR).

#### 2.3.1. Local leg and body coordination at the segment level (LCS)

The millipede-inspired turning approach follows the leg and body coordination of millipedes (Garcia et al., 2015). To implement the same strategy in our robot, we can directly manipulate the movement of each *Tc* joint at each leg to move in synchronization with the body joint activities driven by the BBC (Figure 4A). This is done by directly adapting the weight of the excitatory output connection from the single recurrent neuron to each *Tc* joint based on the body joint activities. In our proposed control system, the orientation changes the body joint in response to changes in sensory inputs from the environment reduce the weights of the excitatory signals sent to the *Tc* motor neurons from large to small values, thereby decreasing the stride length of the first two leg pairs between the body joint undergoing turning. There is an exception for the last two leg pairs connected to the last two segments, which followed the same commands as the leg pair on the final body segment due to a discrepancy between the number of segments and body joints. In real millipedes, the last segments and their legs are usually maintained in their original positions for propagating the body forward, as seen in Figure 9 of Garcia et al. (2015). The LCS control strategy implementation can be found in Supplementary material.

#### 2.3.2. Simultaneous leg amplitude reduction (SAR)

In addition to the significance of LCS, we also designed another strategy (Rosano and Webb, 2006) where the stride length of all legs inside the turning arc is reduced simultaneously (Figure 4B). In contrast to the local changes at the segment level in the previous strategy, when the robot begins to turn right, all the stride lengths of the right legs will be reduced by reducing the weights of the excitatory signals sent to the *Tc* motor neurons. This reduction occurs regardless of the body joint activity at the segment level. The stride length between two leg pairs will be reduced when encountering an obstacle, even if the body joint between the two leg pairs remains in its original position. A distinguishing feature in this control mechanism from the previous one is that the changes in the stride lengths are programmed based on the changes in the sensory inputs from the SPM instead of the body joint signals from the BBC. The SAR control strategy implementation can be found in Supplementary material.

#### 2.3.3. Global leg phase reversion (GPR)

Previously (Mingchinda et al., 2022), the phase of the legs reversed according to the sensory signal changes in response to the presence of an obstacle due to the reversal of the sensory input signals. Thus, the phase reversal of the legs is controlled by the SPM. Therefore, the robot can move backward in a manner similar to Grinke et al. (2015). For example, suppose the millipede robot detects an obstacle on the left side. In that case, all legs on the right side will simultaneously change their movement directions regardless of the behavior of body joints (Figure 4C). These changes in the phase are implemented by reversing the weight sign of the output from single recurrent neuron to each *Tc* joint at each leg to move the *Tc* joint in the opposite direction. There is a direct stride length control as the robot turns, which differentiates this turning strategy from the previous two. This approach is based on a typical spot turning behavior of robots, which is comparable to an engineering control approach. The GPR control strategy implementation can be found in Supplementary material.

## 3. Results

### 3.1. Turning behavior of the millipede-like robot using different leg and body coordination strategies

We described how the integration of CPG-based control with a series of single recurrent neurons produces the direct-wave gait pattern seen in real millipedes (Barnwell, 1965; Garcia et al., 2015, 2020; Ambe and Aoi, 2019; Yasui et al., 2021). Here, the CPG propagates oscillatory output signals to both the *Tc* and *Pf* joints to drive the motion of the legs. In contrast, the SPM propagates the sensory information about the changes in the environment to the *Tc* joints to adapt the robot's walking behavior as it encounters obstacles. To examine the turning behavior of millipede robot which encountering an obstacle, we defined three approaches to leg and body coordination. The first approach described in Section 2.3.1, closely resembles the behavior of the millipedes in the environment (Garcia et al., 2015), whereby the stride length between each leg pair gradually adapts to the changes in the orientation of each body segment. To compare the advantage of this strategy against other leg and body coordination mechanisms, we created a setup in which the robot was required to move away from the 200 cm wall located directly in front of the robot. As the robot moved toward the wall, the sensory outputs from the SPM change as one or both antennae detect the presence of the wall, making the robot turn away from the wall to avoid collision. Using the task involving the wall, we could establish a turning behavior in a manner similar to real millipedes (Garcia et al., 2015).

Coupled with oscillatory signals from the CPG and pre-processed sensory information from the SPM, local control of *Tc* joints using series of single recurrent neurons produced the desired direct wave patterns that mimic those seen in real millipedes. After we established the direct wave gait, we implemented the three turning strategies by having the millipede robot walk against 200 cm wall (Figure 4). Previously, we have found that different *ws* weights led to different turning behavior due to the different sizes of the hysteresis loop generated (Mingchinda et al., 2022). Thus, we varied *ws* from (0.0, ..., 4.0) during the wall task to determine the differences in the turning behaviors when *ws* are different for each of the three turning strategies.

We set the recurrent connection weight (*ws*) in the BBC (Figure 2C) to (0.0, 1.0, ..., 4.0) to test the efficiency of each leg and body coordination strategy during turning away from a wall. As previously determined, the turning angle of the robot was larger as *ws* increased. Our preliminary findings showed that when *ws* = 0.0 and 1.0, the robot instantly collided with the wall as its body bent in a C-shaped fashion (Mingchinda et al., 2022). This is consistent with our previous study, which showed that *ws* must be sufficiently high to produce the adequate hysteresis loop size to sustain the output signals and temporal delays. When *ws* was 0.0 or 1.0, the hysteresis loop size was small, resulting in shorter temporal delays between every single recurrent neuron in the series. As these single recurrent neuron series project the outputs of every tenth neuron to the body joint, the turning angle of each body segment was directly impacted by the size of the *ws* weight.

**Figure 5:**
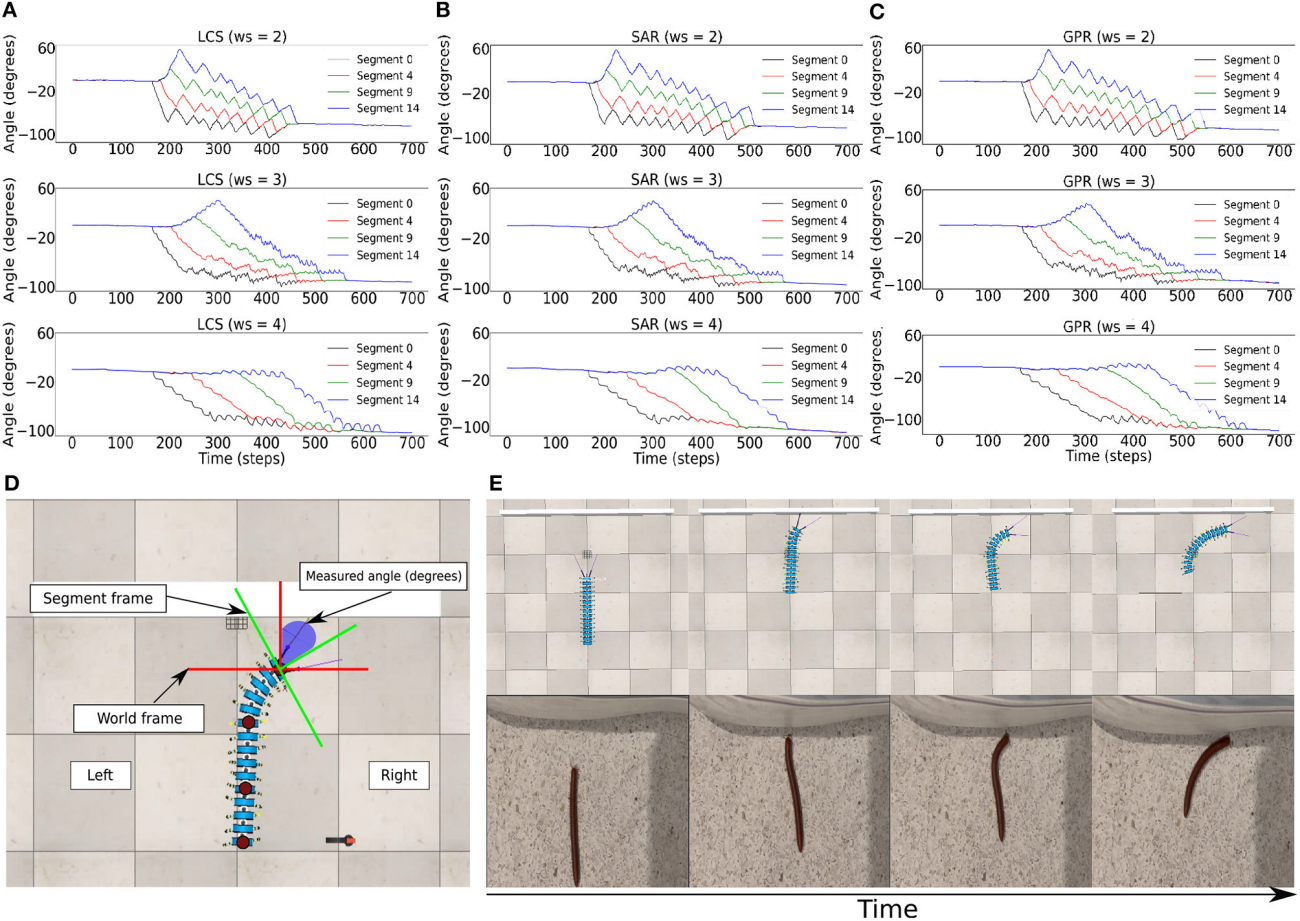
The BBC was used to control the turning behavior of the robot via changing the turning angle size and duration, which was done by changing *ws*. Body segment orientation across *ws* = 2.0, *ws* = 3.0, *ws* = 4.0 in the wall task for **(A)** LCS **(B)** SAR **(C)** GPR, showing every fifth segment in the millipede robot. Here, it is seen that the temporal delays between the turning of each segment are more significant as *ws* is higher due to the greater size of the hysteresis loop. **(D)** The measurement of the orientation of each body segment relative to the world frame inside the simulation during the wall task. **(E)** An example of the turning behavior using the gradual leg amplitude change turning strategy (LCS) compared to a real millipede turning behavior, showing the comparable turning behavior starting from the anterior to posterior segments. A video of the robot turning behavior under all setups can be seen at https://www.manoonpong.com/LBC/video1.mp4.

We implemented the body bending control via the BBC, with *ws* = (2.0, 3.0, 4.0) (Figure 2C), for LCS (Figure 5A), SAR (Figure 5B), and GPR (Figure 5C). The orientation of every fifth body segment was measured by the angle of deviation from the millipede robot's segment frame against the world frame to compare the absolute body segment angles across different *ws* values (Figure 5D). This enabled us to measure the changes in the orientation of the robot during turning against its original position in the world frame. Using the BBC, we manipulated the turning behavior of our robot in terms of both the turning angle and bending duration, which generates the turning behavior comparable to a real millipede (Figure 5E). Owing to a larger hysteresis loop in larger *ws*, both the turning angles of segments and temporal delays are greater (Figure 3C). Notably, larger *ws* leads to a more extended body segment turning time, suggesting higher temporal delays due to a larger hysteresis loop.

However, the differences in the turning performance for avoiding the wall across all three turning conditions are not pronounced at this stage, apart from differences in the *Tc* joint behaviors in Figure 4. We hypothesized that the effect of using different leg and body coordination strategies would become more pronounced as the robot is required to move in a more complex environment, e.g., narrow space. The robot has less opportunity to change the bending direction or rotate its body in narrow spaces, as opposed to the broader room in the wall task. If the time spent by the millipede in a bent position during turning is long, issues can arise when the millipede needs to escape sharp corners and narrow spaces to prevent entrapment.

Thus, we expect that (a) different *ws* will lead to different turning behaviors and performance inside narrow spaces, with larger *ws* producing larger body turning angles, and (b) different body and leg coordination strategies will lead to different turning behavior and performance as well. To expand on the latter, this is because if the robot can reduce the stride length synchronously with the changes in the local body joints at the segment level, it can save time in realigning the activities of all legs where the changes in the sensory input are small. For example, if the robot only needs to make a quick turn, the leg and body coordination at the segment level would imply that the robot does not need to change actions of other leg joints that are irrelevant to making quick turns.

### 3.2. Navigation in narrow environments

**Figure 6:**
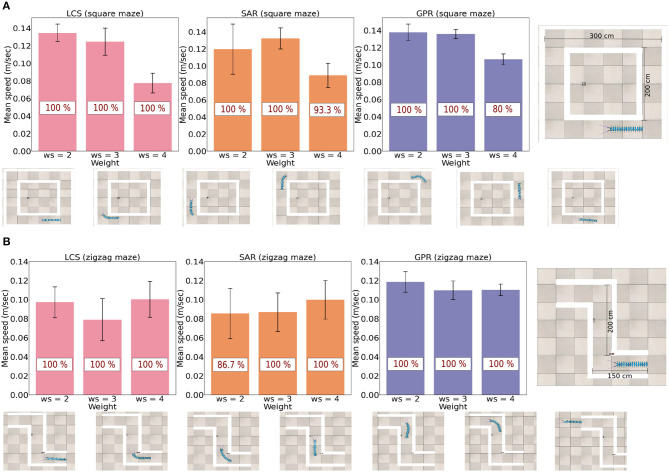
The success rate (in red) and the average speed (m/s) across all three leg and body coordination strategies for *ws* = (2.0, 3.0, 4.0) next to their respective maze type. **(A)** Success rate and average speed for the square maze. **(B)** Success rate and average speed for the zigzag maze. Snapshots exemplify the robot navigation behavior for *ws* = 2.0 of LCS, see also a video of the robot navigation behavior under all setups at https://www.manoonpong.com/LBC/video2.mp4.

A key advantage of real millipedes is the ability to walk through narrow spaces seamlessly. The performance of all three different leg and body bending strategies were tested using the speed of movement and success rates through narrow spaces (Figure 6), where the maze's width was smaller than the total length of the millipede robot, including the sensor range. Two types of narrow environments were used. The zigzag maze requires two turning directions and smaller distances between each corner, while the square maze requires one direction with longer distances between each corner. Consequently, differences exist in the advantages of each leg adapting and body bending strategy between two narrow environments due to the differences in the turning task required. For example, the millipede robot must rebound back to a straight body quicker in a zigzag maze to successfully escape from the maze.

There were fifteen runs per each combination of each leg-body coordination strategy and each *ws* value, where the robot's initial head direction varied from facing the left, forward, and right to avoid potential biases and noises linked to the initial condition. All time measurements were in seconds, inside the simulation. Owing to the different lengths of the walking path, the cut-off point for the failure of the zigzag and square mazes was set to 90 and 200 s, respectively, to leave the maze. Consequently, the success rates of three leg adapting strategies combined with different body bending angles were counted based on the number of completed trials. Finally, speed was measured in (m/s), where only successful trials will be included in the average speed data.

The study found that there are varied rates of success and speed for three turning strategies when *ws* = (2.0, 3.0, 4.0) (Figure 6). It can be seen that *ws* = 4.0 produced notably slower walking speed in comparison to other *ws* weights in the square maze. This is because a large value of *ws* leads to a large hysteresis loop, resulting in a longer body turning time. The extended turning time can lead to overturning and eventually result in a curling behavior if the robot persistently detects a wall in the maze (see Supplementary Figure S1). In fact, there are differences in the ideal *ws* weight for each leg-body coordination strategy. In the less complex square maze with one required turning direction, *ws* = 2.0 (short body turning time) was the most efficient in both measures for LCS and GPR but *ws* = 3.0 was the fastest and most successful *ws* weight for SAR. Among all control strategies, the millipede-like turning strategy LCS and the leg amplitude reversion-based turning strategy GPR at *ws* = 2.0 performed equally well and outperformed other setups.

For the more complex zigzag maze, which requires turning in two directions to escape the environment, LCS with *ws* = 2.0 and *ws* = 4.0 exhibited almost equally excellent performance. GPR with *ws* = 2.0 still demonstrated the best performance compared to other *ws* weights. Interestingly, in this maze, SAR with *ws* = 4.0 showed better performance than with *ws* = 3.0. This suggests that the extended turning time (due to a large *ws* weight) may be advantageous for leg amplitude reduction-based turning control (LCS and SAR). Taken together, different turning strategies exhibit different preferences for the *ws* weight based on measures of average walking speed and success rate. If LCS and GPR are used, a small *ws* weight (short body turning time) is sufficient. However, SAR requires a large *ws* weight (extended body turning time) when turning in more than one direction.

To further investigate the interaction between sensory inputs, walking behavior, and turning behavior, we plotted the activities of the *Tc* joints and body segment orientations during navigation in the square and zigzag mazes. This allowed us to examine the activities of the leg and body joints as the robot employed each of the three leg and body coordination strategies while navigating inside each maze.

**Figure 7:**
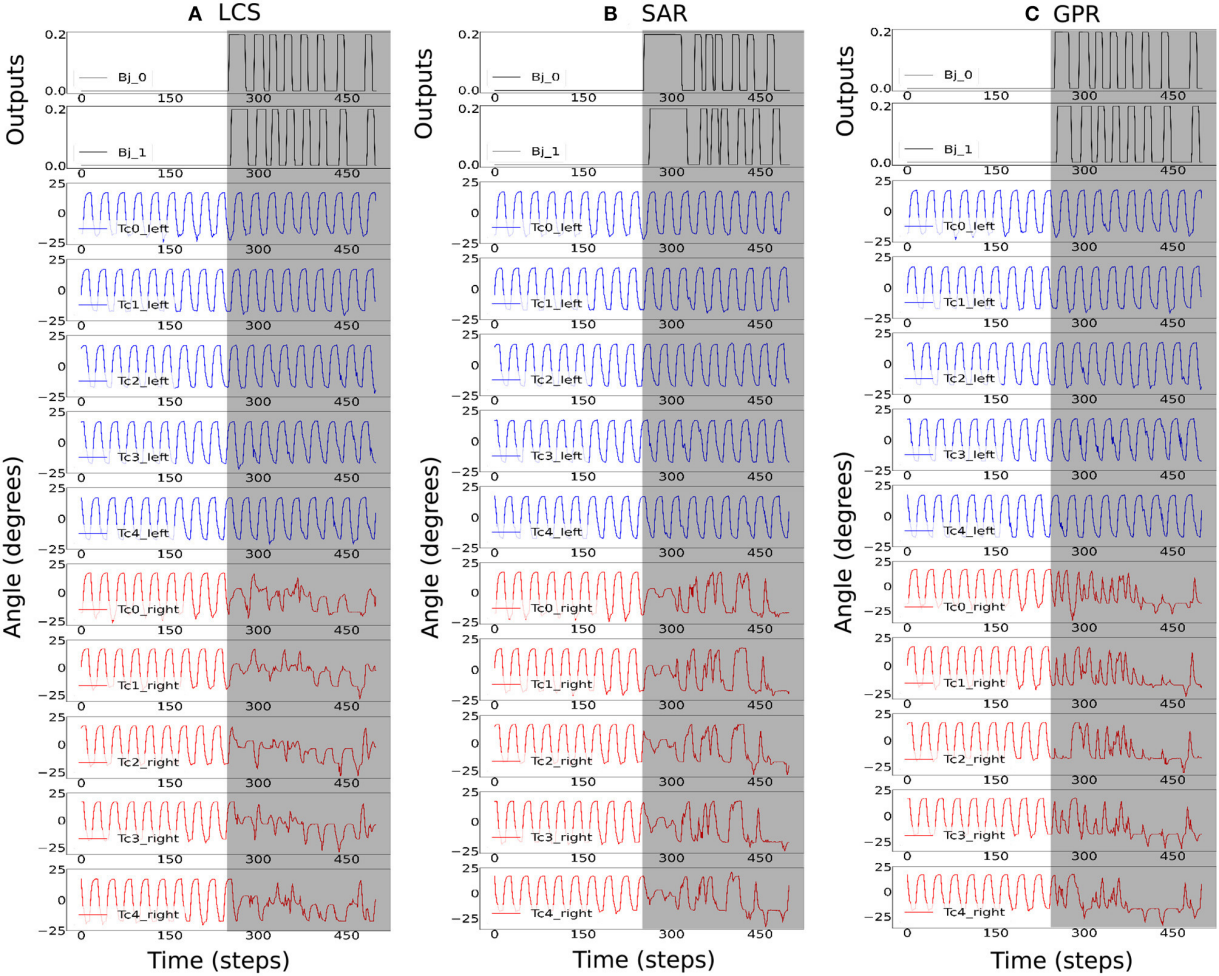
The robot signals during navigating in the square maze environment. The body joint (*Bj*_0_, *Bj*_1_) positions, the joint positions of the first five left *Tc* joints, and the joint positions of the first five right *Tc* joints for *ws* with the highest success rates, where **(A)** *ws* = 2.0 for LCS, **(B)** *ws* = 3.0 for SAR, and **(C)** *ws* = 2.0 for GPR. The gray boxes indicate the period of time that each leg-body coordination strategy was executed as a response to changes in the sensor and/or body signals. Note that the positive body joint signals indicate the right turn. For clarity, the signals were from a period of the square maze navigation.

Figure 7 shows the activities of the first two body joints, along with the first five left and right *Tc* joints as the robot moved across the square maze for the most ideal *ws* weight for LCS (Figure 7A), SAR (Figure 7B), and GPR (Figure 7C). The gray area depicts the period that the robot performed turning. As expected, Figure 7 shows that as the robot turned right, changes in the joint angles were first seen in the first right *Tc* joint, followed by the right *Tc* joints of subsequent legs along the body. This showcases our millipede-like locomotion that mimics the millipedes in nature. The distinguishing feature between LCS and SAR can be observed, where the angles of the *Tc* joints on the right leg changed simultaneously as the body started the turn right in the case of SAR. GPR also showed changes in the *Tc* joints as the robot turned right. Instead of amplitude reductions as seen in LCS and SAR, GPR reversed the *Tc* joint signals.

**Figure 8:**
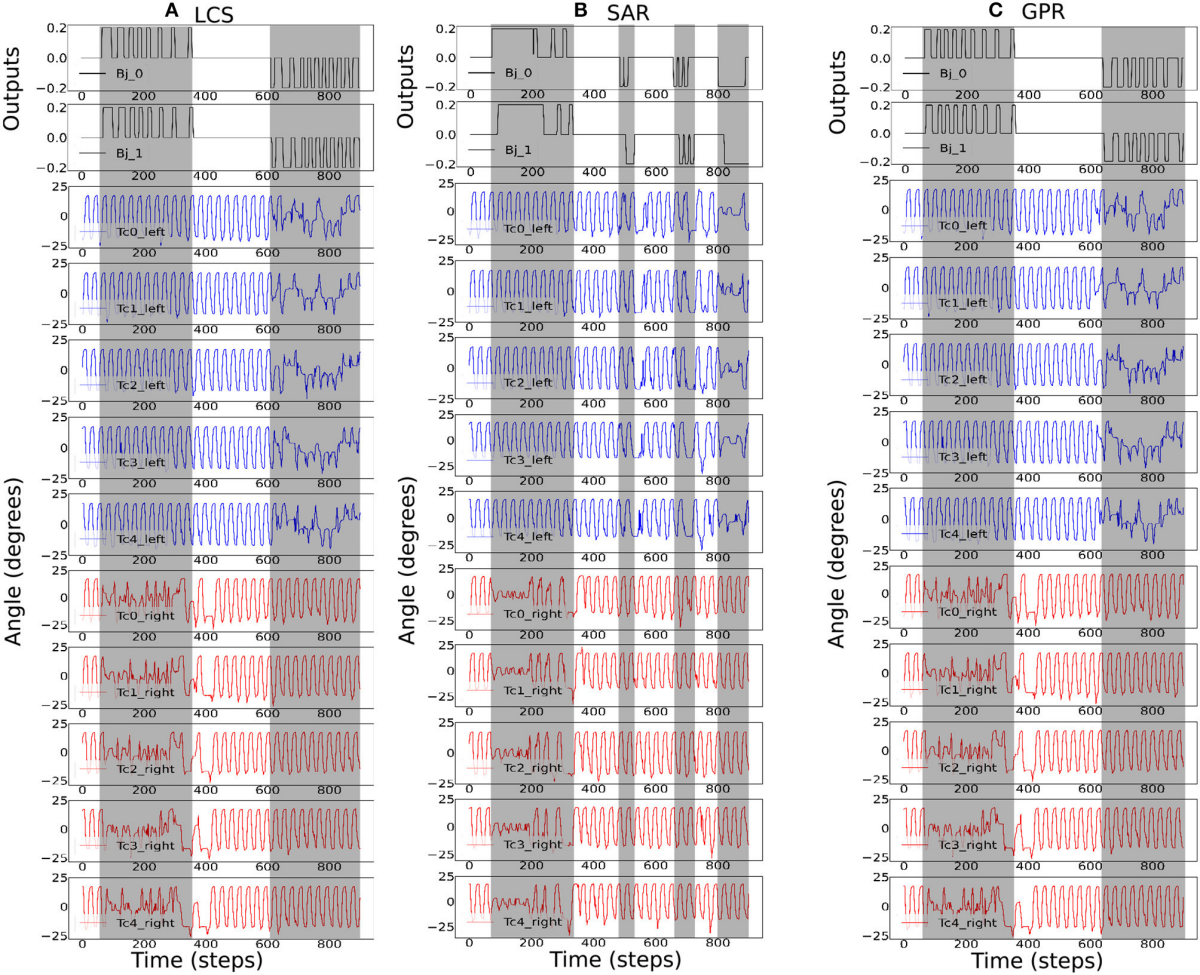
The robot signals during navigating in the zigzag maze environment. The body joint (*Bj*_0_, *Bj*_1_) positions, the joint positions of the first five left *Tc* joints, and the joint positions of the first five right *Tc* joints for *ws* with the highest success rates, where **(A)** *ws* = 2.0 for LCS, **(B)** *ws* = 4.0 for SAR, and **(C)** *ws* = 2.0 for GPR. The gray boxes indicate the period of time that each leg-body coordination strategy was executed as a response to changes in the sensor and/or body signals. Note that the positive and negative body joint signals indicate the right and left turns, respectively. For clarity, the signals were from a period of the zigzag maze navigation.

Figure 8 shows the most ideal *ws* for each turning strategy based on the walking speed and success rate previously shown in Figure 6. As the zigzag maze involved left and right turns, we see positive and negative *Bj* signals showing right and left turns, respectively. As expected, Figure 8A shows the gradual change in the right *Tc* joint angles as the robot turned right for the LCS condition, while SAR (Figure 8B) shows simultaneous right *Tc* angle changes as the body bent. As *ws* was 4.0 for SAR, it can also be observed that the turning time of *Bj* was longer compared to LCS and GPR (Figure 8C). This is consistent to the pattern we found in our previous work (see Figure 3 of Mingchinda et al., 2022). Larger turning angles were also observed in addition to longer turning times for larger *ws*.

Therefore, this study reveals that different *ws*, which is responsible for the control of body bending angle in the BBC, is important for different turning strategies. The larger the *ws* value, the larger the turning angle and temporal delays between each body joint. We also found variations in the ideal *ws* weight across all turning strategies and maze types. One consideration is the difference in the required turning directions between the zigzag and square mazes. The zigzag maze poses the need for the robot to quickly change the turning directions (first turning to the right and then turning to the left). This is in contrast to the square maze, which required turning in only one direction. Thus, the quick changes in the turning direction led to more fluctuations in the IR signals. Consequently, there was more frequent swinging of the body from left to right, and vice versa, compared to the square maze environments.

### 3.3. Navigation in a cluttered environment

**Figure 9:**
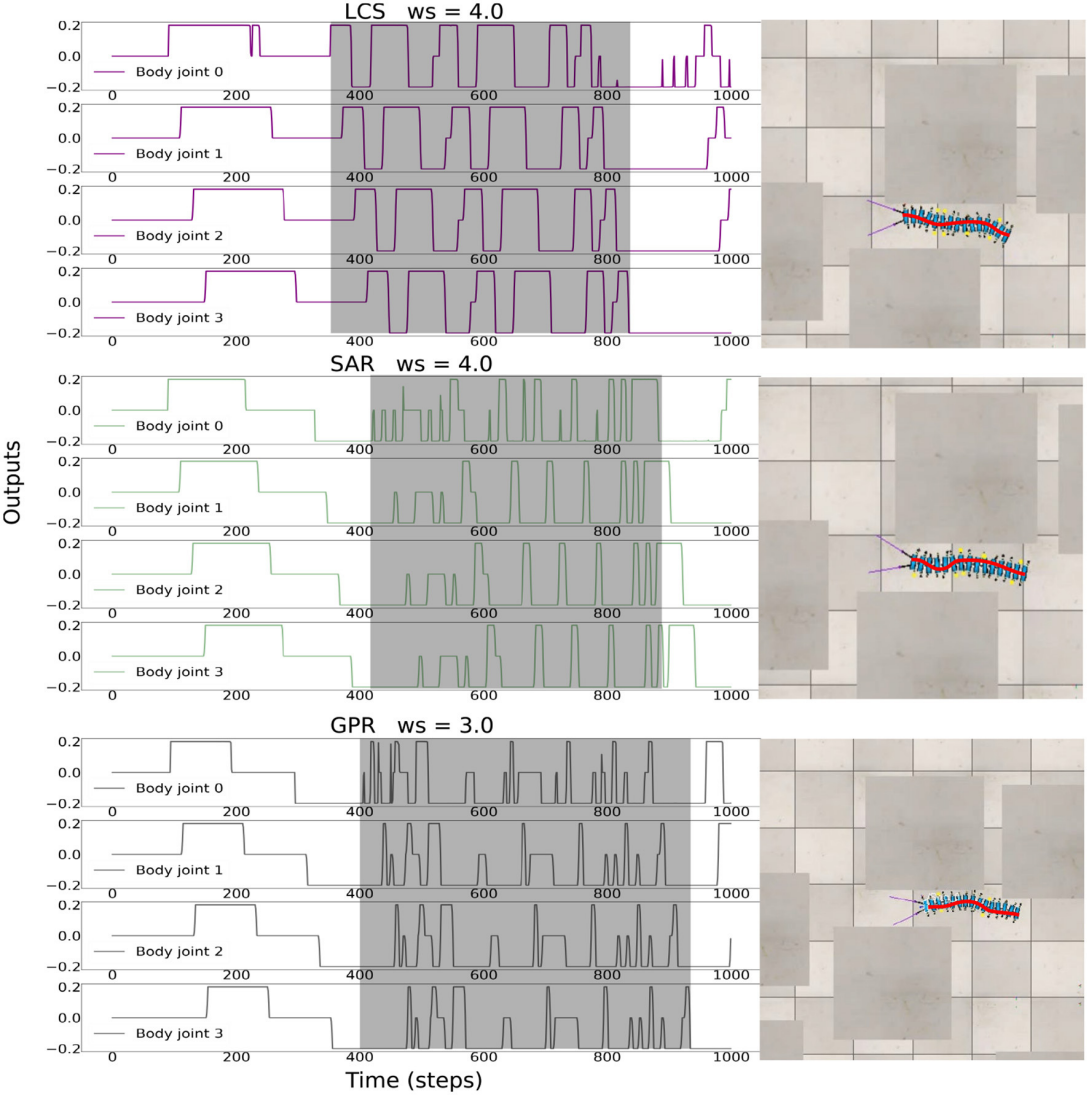
The body undulations under LCS with *ws* = 4.0, SAR with *ws* = 4.0, and GPR with *ws* = 3.0 during the movement of the robot through the complex environment. This represents *ws* that produced the highest success rate per each turning strategy in the complex maze environment. The gray area shows the undulation of the body, where negative body joint outputs leading to left turns, and positive body joint outputs leading to right turns. A video of the robot navigation behavior under all setups can be seen at https://www.manoonpong.com/LBC/video3.mp4.

Finally, we tested all turning strategies against a cluttered environment across all three *ws*, i.e., 2.0, 3.0, and 4.0, of the BBC used. The cluttered environment consisted of objects placed randomly across a scene with no specific required turning directions, as opposed to the zigzag and square mazes. In this environmental setup, there were gaps between obstacles and each gap was made as narrow as the total width of the robot. Interestingly, we observed that all of our control strategies can produce emergent body undulations by rapidly switching body joint output signals between positive and negative values based on sensory feedback from the environment (Figure 9). behaviorally, the robot's body twisted in a wave-like fashion inside narrow gaps within the cluttered environment in order to escape from the maze as a result of rapidly switching sensory feedback from the left and right antennae. We conducted 15 trials for each combination of *ws* and leg-body coordination strategy. For a trial to be considered successful, the millipede's body must be completely clear of obstacles in its exit direction within 90 s. The success rate of each combination is shown in Table 1.

**Table 1:** Comparison table showing the success rate of robot navigation in the cluttered environment under different control strategies (LCS, SAR, and GPR) and different turning angle duration (*ws* = 2.0, 3.0, 4.0).

**Weight**	**LCS**	**SAR**	**GPR**
*ws* = 2.0	7%	13%	27%
*ws* = 3.0	67%	53%	67%
*ws* = 4.0	80%	67%	13%

Overall, *ws* = 3.0 produced the most successful trials across all turning strategies. For *ws* = 2.0, GPR had the most successful trials, followed by SAR and LCS. However, for *ws* = 3.0, LCS produced the tied result with GPR, followed by SAR. Finally, at *ws* = 4.0, LCS had the most successful trials, followed by SAR and GPR. The preference for GPR at *ws* = 2.0 was consistent with our results in Figure 6. GPR with a large weight failed as a result of overturning and the body curling behavior, which caused the robot to get stuck in a narrow gap. LCS seems to reveal a pattern opposite to those in Figure 6. Here, *ws* = 4.0 produced the highest amount of successful trials. Finally, SAR showed preferences for higher body turning angles, similar to our zigzag maze results in Figure 6. Taken together, our findings for narrow space navigation of the millipede-inspired robot indicate that all turning strategies can induce body undulations as a response to rapidly changing sensory information, specifically, when the robot alternatively detects the side walls.

## 4. Discussion

In the previous sections, we described the performance of three different strategies for coordinating the legs and body of the millipede during turning away from an obstacle, e.g., wall. We produced a turning behavior comparable to a real millipede robot (Figure 5E) by implementing our first leg-body coordination strategy (LCS). Here, we aimed to extend our previous findings for achieving a leg and body coordination for the control of turning behavior in a multi-segmented, multi-legged robot. Similar to our previous study, we exploited the neurodynamics of single recurrent neurons connected in series for controlling the gait of the 30-legged millipede robot, along with its body joints. The CPG was used to generate basic rhythmic movement pattern in both the *Tc* and *Pf* joints at each leg, while the SPM was used to filter and process incoming sensory information from the environment before propagating its outputs to the recurrent neurons controlling the *Tc* joints. Similarly, the recurrent neurons controlling the body joints receive inputs from the SPM, which enabled the robot to turn left or right depending on the location of the obstacle. Our main contribution is the implementation of millipede-inspired leg and body coordination during turning by controlling the stride length between each leg pair based on sensory feedback and body segment bending. The millipede-inspired leg and body coordination was compared to two other leg and body coordination strategies, that is, the simultaneous reduction of the stride lengths, and phase reversal of all leg pairs on the opposite side of the obstacle. This was done to explore the uniqueness and potential of the millipede-inspired leg and body coordination as a turning control method for millipede-like robots.

Using a series of recurrent neurons to implement direct-wave gait and local control on the connection weight between the single recurrent neuron—*Tc* motor neuron pair, we could replicate the turning behavior seen in millipedes. Specifically, only the stride lengths of the leg pairs connecting to the bent segments during turning are reduced (Garcia et al., 2015). Moreover, different *ws* at the body joints implemented using the BBC also produced different turning angles in our millipede robot with different temporal delays between anterior and posterior segments. Next, narrow maze navigation tasks were used to explore the efficiency of each leg and body coordination strategy for the turning control of the robot. Taken together, our findings suggest that the optimal *ws* values are different across different turning strategies, as measured using maze completion speed and the success rate.

Upon further observation of slow average walking speed under all conditions or failure in some conditions, we observed that this was due to the curling behavior of the robot (see Supplementary Figure S1). This curling behavior is the result of continuous activation of an IR sensor from one side of the body. This continuous activation in turn leads to continued bending in all body joints, starting from the anterior to posterior segments. As a result, the robot's body curves into a C-shape and may get stuck. In the future, we will develop a control mechanism to avoid this situation.

Previous studies applied the neurodynamics of single recurrent neurons (hysteresis effect) for controlling bio-inspired multi-legged robots (von Twickel et al., 2011; Von Twickel et al., 2012; Grinke et al., 2015; Mingchinda et al., 2022). In line with previous studies, our current study uses the hysteresis effect to modulate the direct-wave pattern of our millipede robot in a manner similar to Kinugasa and Sugimoto (2017) and Homchanthanakul and Manoonpong (2021) by producing temporal delays between each pair of legs connecting to body segments. By directly controlling the leg adaptation during obstacle avoidance tasks using single recurrent neuron activities, we generated three different strategies for leg and body coordination of our millipede robot during turning and avoiding obstacles. We also applied the same properties of recurrent neurons, namely the hysteresis effect, to control the adaptive behavior of each body segment of the robot as it escapes from complex environments. It is worth highlighting that although our investigated control strategies focus on leveraging sensory information and temporal delays (short-term memory) within the neural control circuits, known as neural computation (Manoonpong and Tetzlaff, 2018), for adaptive body-leg coordination, it's also possible to achieve adaptive body-leg coordination through open-loop control with mechanical intelligence, referred to as morphological computation (Thuruthel and Iida, 2022), as demonstrated in Aoi et al. (2016) and Chong et al. (2023). Generally, utilizing sensory information for control becomes more advantageous in situations characterized by significant variations in robot-environment interactions (e.g., navigating in obstacle and narrow space environments shown here), as highlighted in Chong et al. (2023), a key concept underpinning our study.

In our previous study, we used a tripod gait as a walking pattern, where each *Tc* joint reacts to sensory information from the environment through velocity regulating networks (VRNs) in combination with the SPM (Mingchinda et al., 2022). Here, we used series of single recurrent neurons to generate the direct-wave gait by projecting the outputs of every eleventh neurons from the series to each leg, starting from the last pair of legs. This generated a walking pattern that is seen in millipedes in nature (Barnwell, 1965; Garcia et al., 2015, 2020). However, there is a trade off concerning the balance of the millipede robot during turning, as reflected in higher locomotion speed and lower task completion time in the GPR strategy for both the square and zigzag mazes (Figure 6). For flies in the *Drosophilia* family, the tripod gait was optimal for higher walking speed, but the tetrapod gait provided greater walking stability (Strauss and Heisenberg, 1990; Szczecinski et al., 2018). Further, tardigrades also utilized a tripod gait, which enabled them to rapidly traverse across multiple terrains (Nirody et al., 2021). Moreover, the higher speed of GPR can also be atrributed to the inadequate balance of the robot as it walks using the wave gait since the center of mass (COM) of multi-legged robots with sprawling motions, such as our model, is more spread out (Aoi et al., 2016; Grzelczyk et al., 2017). These findings explain the effectiveness of GPR, and important implications for the optimization of millipede robot locomotion in the future. For example, the stability and speed trade off must be considered during the design of the robot depending on several factors such as terrains.

For future studies using hardware to implement locomotion of millipede-like robot in narrow space environments, the following considerations must be taken into account. Firstly, the friction of the ground will influence the stability of the robot during walking. It is necessary to apply certain measures to the feet of the robot in order to prevent slipping. Secondly, the torque of all joints in the legs and body must be high enough as to prevent erratic behaviors in response to both changes in the sensory inputs from the environment, and also to prevent the robot from collapsing too much during walking with the direct-wave gait. Note that in the real world, the body of the millipede moves slightly up and down during walking as the direct wave is propagated through each leg. This means that the ideal level of leg and body joint torques must be further investigated in order to generate the most stable walking behavior without compromising the reaction to the environmental changes. Lastly, if the robot is tasked with exploring environments with limited sensory information, adaptive leg-body coordination control through morphological computation or mechanical intelligence (Chong et al., 2023) should be considered. Thus, combining neural and morphological computation aspects can provide adaptive and robust locomotion of multi-segmented, legged robots for complex, unpredictable real-world environments.

To conclude, our study investigated the two dimensions of the locomotion control of millipede-like robot, namely the leg and body control. We exploited the neurodynamics of recurrent neurons to generate hysteresis effects that produced both the direct-wave gait and body bending mechanism in the BBC. Although LCS produced the walking and turning behaviors most similar to millipedes in nature, we observed that GPR produced the best performance in terms of locomotion speed in narrow environments. As both studies in animals and bio-inspired robotics showed different trade off between each gait pattern, future studies must explore the relationship between different types of gait patterns, including the tripod and tetrapod-like gaits, and how they interact with the body bending mechanism to produce the desired behavior depending on the function of a robot.

## Data availability statement

The raw data supporting the conclusions of this article will be made available by the authors, without undue reservation.

## Author contributions

PM and NM conceived the research idea. NM developed and implemented the control methods, analyzed the data, and wrote the manuscript. PM provided the general direction of the study. PM, VJ, and BL supervised the development of the neural control system, helped with data analysis, and revised the manuscript. All authors contributed to the article and approved the submitted version.

## Footnotes

^1^It is important to note that centipedes employ out-of-phase coordination for contralateral legs, while millipedes use in-phase coordination for contralateral legs (see Figure 1B). While both centipedes and millipedes share similarities in terms of their redundant leg structure and robust locomotion performance, centipedes exhibit faster movement. Furthermore, centipedes naturally display body undulations as they walk (Yasui et al., 2019; Chong et al., 2022); such a feature is not commonly observed in millipedes.

## References

[B1] Akkawutvanich C. Knudsen F. I. Riis A. F. Larsen J. C. Manoonpong P. (2020). Adaptive parallel reflex-and decoupled cpg-based control for complex bipedal locomotion. Rob. Auton. Syst. 134, 103663. 10.1016/j.robot.2020.103663

[B2] Ambe Y. Aoi S. (2019). “Simple multi-legged model reveals that retrograde-wave gait rather attenuates body oscillation than direct-wave gait,” in 3rd International Symposium on Swarm Behavior and Bio-Inspired Robotics (SWARM2019), Vol. 1750 (Okinawa: EasyChair), 3.

[B3] Ambe Y. Aoi S. Konyo M. Tadokoro S. (2022). “Local sensory feedback generates various wave gaits in multi-legged robots via embodied sensorimotor interaction,” in 2022 13th Asian Control Conference (ASCC) (Jeju-do: IEEE), 1379–1383.

[B4] Ambe Y. Aoi S. Tsuchiya K. Matsuno F. (2021). Generation of direct-, retrograde-, and source-wave gaits in multi-legged locomotion in a decentralized manner via embodied sensorimotor interaction. Front. Neural Circuits 15, 706064. 10.3389/fncir.2021.706064 34552472 PMC8450536

[B5] Aoi S. Manoonpong P. Ambe Y. Matsuno F. Wörgötter F. (2017). Adaptive control strategies for interlimb coordination in legged robots: a review. Front. Neurorobot. 11, 39. 10.3389/fnbot.2017.00039 28878645 PMC5572352

[B6] Aoi S. Tanaka T. Fujiki S. Funato T. Senda K. Tsuchiya K. (2016). Advantage of straight walk instability in turning maneuver of multilegged locomotion: a robotics approach. Sci. Rep. 6, 1–10. 10.1038/srep30199 27444746 PMC4957114

[B7] Avirovik D. Butenhoff B. Priya S. (2014). Millipede-inspired locomotion through novel u-shaped piezoelectric motors. Smart Mater. Struct. 23, 037001. 10.1088/0964-1726/23/3/037001

[B8] Avirovik D. Priya S. (2013). “Crawling-inspired robot utilizing l-shape piezoelectric actuators,” in 2013 IEEE/ASME International Conference on Advanced Intelligent Mechatronics (Wollongong, NSW: IEEE), 894–899.

[B9] Bai L. Hu H. Chen X. Sun Y. Ma C. Zhong Y. (2019). Cpg-based gait generation of the curved-leg hexapod robot with smooth gait transition. Sensors 19, 3705. 10.3390/s19173705 31455002 PMC6749326

[B10] Barnwell F. H. (1965). An angle sense in the orientation of a millipede. Biol. Bull. 128, 33–50. 10.2307/1539387

[B11] Chen J. San H. Wu X. (2019). Gait regulation of a bionic quadruped robot with antiparallelogram leg based on cpg oscillator. Complexity 2019, 1–11. 10.1155/2019/5491298

[B12] Chong B. Aydin Y. O. Rieser J. M. Sartoretti G. Wang T. Whitman J.. (2022). A general locomotion control framework for multi-legged locomotors. Bioinspir. Biomimetics 17, 046015. 10.1088/1748-3190/ac6e1b 35533656

[B13] Chong B. He J. Soto D. Wang T. Irvine D. Blekherman G.. (2023). Multilegged matter transport: a framework for locomotion on noisy landscapes. Science 380, 509–515. 10.1126/science.ade4985 37141349

[B14] Enghoff H. (1992). “The Size of a Millipede. Berichte der naturhistorisch-medizinischen Vereins Innsbruck,” in 8th International Congress of Myriapodology (Innsbruck).

[B15] Francisco A. Nocelli R. C. Fontanetti C. S. (2015). The nervous system of the neotropical millipede gymnostreptus olivaceus schubart, 1944 (spirostreptida, spirostreptidae) shows an additional cell layer. Anim. Biol. 65, 133–150. 10.1163/15707563-00002466

[B16] Fukui T. Fujisawa H. Otaka K. Fukuoka Y. (2019). Autonomous gait transition and galloping over unperceived obstacles of a quadruped robot with cpg modulated by vestibular feedback. Robot. Auton. Syst. 111, 1–19. 10.1016/j.robot.2018.10.002

[B17] Garcia A. Krummel G. Priya S. (2020). Fundamental understanding of millipede morphology and locomotion dynamics. Bioinspir. Biomimetics 16, 026003. 10.1088/1748-3190/abbdcc 33007767

[B18] Garcia A. Priya S. Marek P. (2015). “Understanding the locomotion and dynamic controls for millipedes: part 1—kinematic analysis of millipede movements,” in Smart Materials, Adaptive Structures and Intelligent Systems, Vol. 57304 (Colorado: American Society of Mechanical Engineers), V002T06A005.

[B19] Grinke E. Tetzlaff C. Wörgötter F. Manoonpong P. (2015). Synaptic plasticity in a recurrent neural network for versatile and adaptive behaviors of a walking robot. Front. Neurorobot. 9, 11. 10.3389/fnbot.2015.00011 26528176 PMC4602151

[B20] Grzelczyk D. Stanczyk B. Awrejcewicz J. (2017). Kinematics, dynamics and power consumption analysis of the hexapod robot during walking with tripod gait. Int. J. Struct. Stabil. Dyn. 17, 1740010. 10.1142/S0219455417400107

[B21] Grzelczyk D. Szymanowska O. Awrejcewicz J. (2019). Kinematic and dynamic simulation of an octopod robot controlled by different central pattern generators. J. Syst. Control Eng. 233, 400–417. 10.1177/0959651818800187

[B22] He B. Si Y. Wang Z. Zhou Y. (2019). Hybrid cpg-fri dynamic walking algorithm balancing agility and stability control of biped robot. Auton. Robots 43, 1855–1865. 10.1007/s10514-019-09839-2

[B23] Hembree D. I. (2009). Neoichnology of burrowing millipedes: linking modern burrow morphology, organism behavior, and sediment properties to interpret continental ichnofossils. Palaios 24, 425–439. 10.2110/palo.2008.p08-098r

[B24] Hoffman K. L. Wood R. J. (2011). “Passive undulatory gaits enhance walking in a myriapod millirobot,” in 2011 IEEE/RSJ International Conference on Intelligent Robots and Systems (IEEE), 1479–1486.

[B25] Homchanthanakul J. Manoonpong P. (2021). Continuous online adaptation of bioinspired adaptive neuroendocrine control for autonomous walking robots. IEEE Transact/ Neural Netw. Learn. Syst. 33, 1833–1845. 10.1109/TNNLS.2021.3119127 34669583

[B26] Hülse M. Wischmann S. Manoonpong P. Twickel A. V. Pasemann F. (2007). “Dynamical systems in the sensorimotor loop: on the interrelation between internal and external mechanisms of evolved robot behavior,” in 50 Years of Artificial Intelligence, eds Lungarella M. Iida F. Bongard J. Pfeifer R. (Springer), 186–195.

[B27] Joly F.-X. Coq S. Coulis M. David J.-F. Hättenschwiler S. Mueller C. W.. (2020). Detritivore conversion of litter into faeces accelerates organic matter turnover. Commun. Biol. 3, 1–9. 10.1038/s42003-020-01392-4 33177652 PMC7658975

[B28] Kano T. Sakai K. Yasui K. Owaki D. Ishiguro A. (2017). Decentralized control mechanism underlying interlimb coordination of millipedes. Bioinspir. Biomimetics 12, 036007. 10.1088/1748-3190/aa64a5 28375850

[B29] Kinugasa T. Sugimoto Y. (2017). Dynamically and biologically inspired legged locomotion: a review. J. Robot. Mechatr. 29, 456–470. 10.20965/jrm.2017.p0456

[B30] Kuroda S. Kunita I. Tanaka Y. Ishiguro A. Kobayashi R. Nakagaki T. (2014). Common mechanics of mode switching in locomotion of limbless and legged animals. J. R. Soci. Interface 11, 20140205. 10.1098/rsif.2014.0205 24718452 PMC4006264

[B31] Long G. Anderson J. Borenstein J. (2002). “The kinematic design of the omnipede: a new approach to obstacle traversion,” in Proceedings 2002 IEEE International Conference on Robotics and Automation (Cat. No. 02CH37292), Vol. 1 (Washington, DC: IEEE), 714–719.

[B32] Ma F. Yan W. Chen L. Cui R. (2022). Cpg-based motion planning of hybrid underwater hexapod robot for wall climbing and transition. IEEE Robot. Automat. Lett. 7, 12299–12306. 10.1109/LRA.2022.3216233

[B33] Manoonpong P. Parlitz U. Wörgötter F. (2012). Internal forward models with efference copies for state estimations in adaptive hexapod locomotion. Front. Comput. Neurosci. 10.3389/conf.fncom.2012.55.00209

[B34] Manoonpong P. Pasemann F. Kolodziejski C. Wörgötter F. (2010). Designing simple nonlinear filters using hysteresis of single recurrent neurons for acoustic signal recognition in robots. In International Conference on Artificial Neural Networks (Springer), 374–383

[B35] Manoonpong P. Pasemann F. Roth H. (2007). Modular reactive neurocontrol for biologically inspired walking machines. Int. J. Rob. Res. 26, 301–331. 10.1177/0278364906076263

[B36] Manoonpong P. Pasemann F. Wörgötter F. (2008). Sensor-driven neural control for omnidirectional locomotion and versatile reactive behaviors of walking machines. Robot. Auton. Syst. 56, 265–288. 10.1016/j.robot.2007.07.004

[B37] Manoonpong P. Tetzlaff C. (2018). Editorial: neural computation in embodied closed-loop systems for the generation of complex behavior: from biology to technology. Front. Neurorobot. 12:53. 10.3389/fnbot.2018.00053 30214405 PMC6125336

[B38] Marek P. E. Buzatto B. A. Shear W. A. Means J. C. Black D. G. Harvey M. S.. (2021). The first true milliped—1306 legs long. Sci. Rep. 11, 1–8. 10.1038/s41598-021-02447-0 34916527 PMC8677783

[B39] Miguel-Blanco A. Manoonpong P. (2020). General distributed neural control and sensory adaptation for self-organized locomotion and fast adaptation to damage of walking robots. Front. Neural Circ. 14, 46. 10.3389/fncir.2020.00046 32973461 PMC7461994

[B40] Mingchinda N. Jaiton V. Leung B. Manoonpong P. (2022). “Neural body bending control with temporal delays for millipede-like turning behaviour of a multi-segmented, legged robot,” in International Conference on Simulation of Adaptive Behavior (Cergy-Pontoise: Springer), 52–63.

[B41] Nirody J. A. Duran L. A. Johnston D. Cohen D. J. (2021). Tardigrades exhibit robust interlimb coordination across walking speeds and terrains. Proc. Nat. Acad. Sci. U. S. A. 118, e2107289118. 10.1073/pnas.2107289118 34446560 PMC8536314

[B42] Oldham K. Rhee C.-H. Ryou J.-H. Polcawich R. Pulskamp J. (2009). “Lateral thin-film piezoelectric actuators for bio-inspired micro-robotic locomotion,” in International Design Engineering Technical Conferences and Computers and Information in Engineering Conference, Vol. 49033 (San Diego, CA), 759–768.

[B43] Ouyang W. Chi H. Pang J. Liang W. Ren Q. (2021). Adaptive locomotion control of a hexapod robot via bio-inspired learning. Front. Neurorobot. 15, 627157. 10.3389/fnbot.2021.627157 33574748 PMC7870720

[B44] Pasemann F. (1993a). Discrete dynamics of two neuron networks. Open Syst. Inf. Dyn. 2, 49–66. 10.1007/BF02228971

[B45] Pasemann F. (1993b). Dynamics of a single model neuron. Int. J. Bifurc. Chaos 3, 271–278. 10.1142/S0218127493000210

[B46] Pasemann F. (1997). A simple chaotic neuron. Phys. D Nonlinear Phenomena 104, 205–211. 10.1016/S0167-2789(96)00239-4

[B47] Reboleira A. S. P. Enghoff H. (2018). First continental troglobiont cylindroiulus millipede (diplopoda, julida, julidae). Zookeys 795, 93–103. 10.3897/zookeys.795.27619 30473610 PMC6237897

[B48] Rohmer E. Singh S. P. Freese M. (2013). “V-rep: a versatile and scalable robot simulation framework,” in 2013 IEEE/RSJ International Conference on Intelligent Robots and Systems (Tokyo: IEEE), 1321–1326.

[B49] Rosano H. Webb B. (2006). “The control of turning in real and simulated stick insects,” in From Animals to Animats 9: 9th International Conference on Simulation of Adaptive Behavior, SAB 2006, Rome, Italy, September 25-29, 2006. Proceedings 9 (Springer), 150–161.

[B50] Shao Q. Dong X. Lin Z. Tang C. Sun H. Liu X.-J.. (2022). Untethered robotic millipede driven by low-pressure microfluidic actuators for multi-terrain exploration. IEEE Robot. Automat. Lett. 7, 12142–12149. 10.1109/LRA.2022.3213137

[B51] Spinello D. Fattahi J. S. (2017). Peristaltic wave locomotion and shape morphing with a millipede inspired system. J. Nonlinear Sci. 27, 1093–1119. 10.1007/s00332-017-9372-7

[B52] Strauss R. Heisenberg M. (1990). Coordination of legs during straight walking and turning in drosophila melanogaster. J. Comp. Physiol. A 167, 403–412. 10.1007/BF00192575 2121965

[B53] Szczecinski N. S. Bockemühl T. Chockley A. S. Büschges A. (2018). Static stability predicts the continuum of interleg coordination patterns in drosophila. J. Exp. Biol. 221, jeb189142. 10.1242/jeb.189142 30274987

[B54] Thuruthel T. G. Iida F. (2022). “Morphological computation and control complexity,” in IOP Conference Series: Materials Science and Engineering, Vol. 1261 (Rome: IOP Publishing), 012011.

[B55] Venkiteswaran V. K. Samaniego L. F. P. Sikorski J. Misra S. (2019). Bio-inspired terrestrial motion of magnetic soft millirobots. IEEE Robot. Automat. Lett. 4, 1753–1759. 10.1109/LRA.2019.2898040

[B56] Venkiteswaran V. K. Tan D. K. Misra S. (2020). Tandem actuation of legged locomotion and grasping manipulation in soft robots using magnetic fields. Extreme Mech. Lett. 41, 101023. 10.1016/j.eml.2020.101023

[B57] von Twickel A. Büschges A. Pasemann F. (2011). Deriving neural network controllers from neuro-biological data: implementation of a single-leg stick insect controller. Biol. Cybern. 104, 95–119. 10.1007/s00422-011-0422-1 21327828

[B58] Von Twickel A. Hild M. Siedel T. Patel V. Pasemann F. (2012). Neural control of a modular multi-legged walking machine: simulation and hardware. Robot. Auton. Syst. 60, 227–241. 10.1016/j.robot.2011.10.006

[B59] Wang B. Zhang K. Yang X. Cui X. (2020). The gait planning of hexapod robot based on cpg with feedback. Int. J. Adv. Robot. Syst. 17, 1729881420930503. 10.1177/1729881420930503

[B60] Wang H. Zhu Z. Jin H. Wei R. Bi L. Zhang W. (2022). Magnetic soft robots: design, actuation, and function. J. Alloys Cpd 922, 166219. 10.1016/j.jallcom.2022.166219

[B61] Wu X. Ma S. (2013). Neurally controlled steering for collision-free behavior of a snake robot. IEEE Transact. Control Syst. Technol. 21, 2443–2449. 10.1109/TCST.2012.2237519

[B62] Yao C. Liu C. Xia L. Liu M. Chen Q. (2022). Humanoid adaptive locomotion control through a bioinspired cpg-based controller. Robotica 40, 762–779. 10.1017/S0263574721000795

[B63] Yasui K. Kano T. Kuroda S. Aonuma H. Hayase Y. Kobayashi R.. (2021). “On the determinant of gait patterns in myriapod locomotion,” in The 9.5th international symposium on Adaptive Motion of Animals and Machines (Ottawa, ON: Virtual Platform).

[B64] Yasui K. Kano T. Standen E. Aonuma H. Ijspeert A. J. Ishiguro A.. (2019). Decoding the essential interplay between central and peripheral control in adaptive locomotion of amphibious centipedes. Sci. Rep. 9, 18288. 10.1038/s41598-019-53258-3 31792255 PMC6889372

[B65] Yasui K. Sakai K. Kano T. Owaki D. Ishiguro A. (2017). Decentralized control scheme for myriapod robot inspired by adaptive and resilient centipede locomotion. PLoS ONE 12, e0171421. 10.1371/journal.pone.0171421 28152103 PMC5289600

[B66] Zhang Y. Wang H. Ding Y. Hou B. (2021). Adaptive walking control for a quadruped robot on irregular terrain using the complex-valued cpg network. Symmetry 13, 2090. 10.3390/sym13112090

