# Supplementary material for: Leg-body coordination strategies for obstacle avoidance and narrow space navigation of multi-segmented, legged robots
Source: Front Neurorobot. 2023 Nov 8;17:1214248. doi: 10.3389/fnbot.2023.1214248 (PMC10663368; doi:10.3389/fnbot.2023.1214248)
Supplement: Supplementary file 1 [file Data_Sheet_1.pdf]

frontiers

Supplementary Information for Leg-body coordination strategies for obstacle avoidance and narrow space navigation of multi-segmented, legged robots

1 DIRECT-WAVE GAIT CONTROL

For generating a direct-wave gait, all neurons in a series of single recurrent neurons for controlling Tc and Pf are realised using discrete-time dynamics. For Tc control, the activation of each single recurrent neuron in the left/right series is described as:

$$a_{i_{L,\ Tc\ Series}}(t) = \begin{cases} (W_{ii}o_{i_{L,Tc\ Series}}(t-1)) + (W_{iC_1}C_1(t)I_1(t)), & i = 0 \\ (W_{ii}o_{i_{L,Tc\ Series}}(t-1)) + (W_{ij}o_{j_{L,Tc\ series}}(t)), i = 1, 2, ..., 155, j = i-1 \end{cases} \tag{S1}$$

$$a_{i_{R,\ Tc\ Series}}(t) = \begin{cases} (W_{ii}o_{i_{R,Tc\ Series}}(t-1)) + (W_{iC_1}C_1(t)I_0(t)), & i = 0 \\ (W_{ii}o_{i_{R,Tc\ Series}}(t-1)) + (W_{ij}o_{j_{R,Tc\ series}}(t)), i = 1, 2, ..., 155, j = i-1 \end{cases} \tag{S2}$$

where $a_{i_{L,\ Tc\ Series}}$ and $a_{i_{R,\ Tc\ Series}}$ refer to activations of all neurons in the left and right Tc series, respectively. W_{ii} refers to the recurrent weight of a single neuron with a value of 1.5, and W_{ij} refers to the inter-neuron weight from neurons j to i with a value of 2.5. For the first neuron of the series ($i = 0$), I_1 and I_0 stand for the outputs from the right and left neurons of the SPM. C_1 stands for the output from the right neuron of the CPG (Fig. 2(a)), and W_{iC_1} stands for the connection weight between I_1 and the first Tc joint with a constant weight of -3.0. The second single recurrent neuron output in the series $a_{i_{L,\ Tc\ Series}}$ and $a_{i_{R,\ Tc\ Series}}$ is projected to the first posterior leg pair to mitigate the effect of potential differences in the signal pattern. After the first posterior leg pair, each subsequent pair receives input from every eleventh neuron in the left or right single recurrent neuron series as described by the second line of Equations (S1), (S2). $o_{i_{L,Tc\ Series}}$ and $o_{i_{R,Tc\ Series}}$ refer to the outputs of recurrent neurons in the left and right Tc series, and $o_{j_{L,Tc\ Series}}$ and $o_{j_{R,Tc\ Series}}$ refer to the outputs of their previous neurons. The outputs of all neurons are calculated using a hyperbolic tangent ($tanh$) activation function ($o_i = tanh(a_i) \in [-1, 1]$).

Equations (S3), (S4) describe the projection of outputs from every eleventh left and right recurrent neurons from the series to left and right Tc motor neurons for driving the Tc joints. The activations of recurrent neurons in two series controlling the left and right Tc joints are described as:

$$a_{k_{L,\ Tc\ Motor}}(t) = W_{k_{L,\ Tc\ Motor}}o_{i_{L,Tc\ Series}}(t), \qquad k = 14, 13, ..., 1, 0, i = 1, 12, ..., 155 \tag{S3}$$

$$a_{k_{R,\ Tc\ Motor}}(t) = W_{k_{R,\ Tc\ Motor}}o_{i_{R,Tc\ Series}}(t), \qquad k = 14, 13, ..., 1, 0, i = 1, 12, ..., 155 \tag{S4}$$

where $a_{k_{L,\ Tc\ Motor}}$ and $a_{k_{R,\ Tc\ Motor}}$ refer to activations of all motors in the left and right Tc joints, respectively. $W_{k_{L,\ Tc\ Motor}}$ is the inter-neuron weight between each left Tc motor and a recurrent neuron in the left series controlling the corresponding Tc joint with a value of 0.3. $W_{k_{R,\ Tc\ Motor}}$ is the inter-neuron weight between each right Tc motor and a recurrent neuron in the right series controlling the corresponding Tc joint with a value of 0.3. $o_{i_{L,Tc\ Series}}$ and $o_{i_{R,Tc\ Series}}$ refer to the outputs of the recurrent neurons in the left and right Tc series, respectively. The outputs of the motor neurons (k) are calculated by a linear activation function.

For Pf control, the activation of each single recurrent neuron in the left/right series is described as:

$$a_{i_{L,\ Pf\ Series}}(t) = \begin{cases} (W_{ii}o_{i_{L,Pf\ Series}}(t-1)) + (W_{iC_0}C_0(t)), & i = 0 \\ (W_{ii}o_{i_{L,Pf\ Series}}(t-1)) + (W_{ij}o_{j_{L,Pf\ series}}(t)), & i = 1, 2, ..., 155, j = i - 1 \end{cases} \tag{S5}$$

$$a_{i_{R,\ Pf\ Series}}(t) = \begin{cases} (W_{ii}o_{i_{R,Pf\ Series}}(t-1)) + (W_{iC_0}C_0(t)), & i = 0 \\ (W_{ii}o_{i_{R,Pf\ Series}}(t-1)) + (W_{ij}o_{j_{R,Pf\ series}}(t)), & i = 1, 2, ..., 155, j = i - 1 \end{cases} \tag{S6}$$

where $a_{i_{L,\ Pf\ Series}}$ and $a_{i_{R,\ Pf\ Series}}$ refer to activations of all neurons in the left and right Pf series, respectively. W_{ii} refers to the recurrent weight of a single neuron with a value of 1.5. W_{ij} refers to the inter-neuron weight of 2.5 from neurons j to i. For the first neuron of the series ($i = 0$), C_0 stands for the output from the left neuron of the CPG (Fig. 2(a)). W_{iC_0} stands for the connection weight between C_0 and the first Pf motor neuron with a constant weight of -2.0. Similar to the Tc joint control, each subsequent pair receives input from every eleventh neuron in the left or right single recurrent neuron series as described by the second line of Equations (S5), (S6). $o_{i_{L,Pf\ Series}}$ and $o_{i_{R,Pf\ Series}}$ refer to the outputs of a recurrent neuron in the left and right Tc series, and $o_{j_{L,Pf\ Series}}$ and $o_{j_{R,Pf\ Series}}$ refer to the outputs of their previous neurons. The outputs of all neurons are calculated using a hyperbolic tangent ($tanh$) activation function ($o_i = tanh(a_i) \in [-1, 1]$).

Equation (S7), (S8) describe the projection of outputs from every eleventh left and right recurrent neuron from the series to left and right Pf motor neurons for driving the Pf joints. The activations of recurrent neurons in two series controlling the left and right Pf joints are described as:

$$a_{k_{L,\ Pf\ Motor}}(t) = W_{k_{L,\ Pf\ Motor}}o_{i_{L,Pf\ Series}}(t), \qquad k = 14, 13, ..., 1, 0, i = 1, 12, ..., 155 \tag{S7}$$

$$a_{k_{R,\ Pf\ Motor}}(t) = W_{k_{R,\ Pf\ Motor}}o_{i_{R,Pf\ Series}}(t), \qquad k = 14, 13, ..., 1, 0, i = 1, 12, ..., 155 \tag{S8}$$

where $a_{k_{L,\ Pf\ Motor}}$ and $a_{k_{R,\ Pf\ Motor}}$ refer to activations of all motors in the left and right Pf joints, respectively. $W_{k_{L,\ Pf\ Motor}}$ is the inter-neuron weight between each left Pf motor and a recurrent neuron in the left series controlling the corresponding Pf joint with a value of 0.3. $W_{k_{R,\ Pf\ Motor}}$ is the inter-neuron weight between each right Pf motor and a recurrent neuron in the right series controlling the corresponding Pf joint with a value of 0.3. $o_{i_{L,Pf\ Series}}$ and $o_{i_{R,Pf\ Series}}$ refer to the outputs of recurrent neurons in the left

and right Pf series, respectively. The outputs of the motor neurons (k) are calculated by a linear activation function.

2 BODY BENDING CONTROL

The body bending control (BBC) drives both the left and right turning motions of the robot via the following equations:

$$a_{L_i}(t) = \begin{cases} 3 \times o_{L_i}(t), & i = 0 \\ ws \times o_{L_i}(t-1) + 3 \times o_{L_j}(t), & i = 1, 2, ..., 130, j = i - 1 \end{cases} \tag{S9}$$

$$a_{R_i}(t) = \begin{cases} 3 \times o_{R_i}(t), & i = 0 \\ ws \times o_{R_i}(t-1) + 3 \times o_{R_j}(t), & i = 1, 2, ..., 130, j = i - 1 \end{cases} \tag{S10}$$

$$a_{Bj_k}(t) = 0.1 o_{L_i}(t) - 0.1 o_{R_i}(t), \qquad k = 0, 1, ..., 13,\ i = 0, 10, ..., 130 \tag{S11}$$

where the activation of neurons controlling left turn a_{L_i} is the sum of the product between each ws weight and the neuron output o_{L_i} , and the output o_{L_j} times the inter-neuron weight between neurons i and j at a constant value of 3. For the activation of neurons controlling right turn, a_{R_i} is the sum of the product between each ws weight and the neuron output o_{R_i} , and the output o_{R_j} times the inter-neuron weight between neurons i and j at a constant value of 3. Finally, the left and right outputs are combined for final body joint control (Equation S11), where a_{Bj_k} stands for the activation of each body joint (Bj_k) as a function of the outputs from the left (o_{L_i}) and right (o_{R_i}) series, with 0.1 as a scaling weight to prevent collision between each body segment during turning. Also, the ws weights at the first left (o_{L_0}) and right (o_{R_0}) neurons were set to 0, to prevent delays in the initial turning behaviour as the robot detects an obstacle. Afterwards, each Bj_k receives inputs from every tenth neuron in the left and right series. The outputs of neurons are calculated using a hyperbolic tangent ($tanh$) activation function ($o_{L_i,R_i} = tanh(a_{L_i,R_i}) \in [-1, 1]$), except for the motor neurons (k) which are calculated linear activation function.

3 LCS CONTROL STRATEGY IMPLEMENTATION

The LCS manipulates the weight of the connection between the recurrent neuron in Tc series depending on the changes in the orientation of the body joint Bj_k:

$$W_{k_{L,\ Tc\ Motor}}(t) = \begin{cases} 0.06, & Bj_k < 0,\ k = 0, 1, \ldots, 13, \\ 0.3, & Bj_k = 0,\ k = 0, 1, \ldots, 13, \end{cases} \tag{S12}$$

$$W_{k_{R,\ Tc\ Motor}}(t) = \begin{cases} 0.06, & Bj_k > 0,\ k = 0, 1, \ldots, 13, \\ 0.3, & Bj_k = 0,\ k = 0, 1, \ldots, 13, \end{cases} \tag{S13}$$

where $W_{k_{R,\ Tc\ Motor}}$ is the weight between the right Tc joint and a recurrent neuron on the right Tc series. $W_{k_{L,\ Tc\ Motor}}$ is the weight between the left Tc joint and a recurrent neuron on the left Tc series. Once a body joint turns, $W_{k_{R,\ Tc\ Motor}}$ will be reduced from 0.3 to 0.06 during the right body turning,

and $W_{k_{L,\ Tc\ Motor}}$ will be reduced from 0.3 to 0.06 during the left body turning. This means that its angle shifted from 0 to a negative value (left turn) or positive value (right turn). As the robot turns by first moving the anterior body segments, the changes in $W_{k_{R,\ Tc\ Motor}}$ or $W_{k_{L,\ Tc\ Motor}}$ will also start from anterior to posterior pairs of legs. Note that, in the robot, there are 14 (0 – 13) Bj joints. However, in Equations (S12), (S13), the Bj joints are up to 15 (0 – 14), which we set as $Bj_{14} = Bj_{13}$. Hereby, we define Bj_{14} as a virtual joint to make the equations to be true and general for all. This means that the feedback signal from Bj_{13} will be used to control Tc_{13} (both left and right) and Tc_{14} (both left and right) through modulating $W_{13_{L,TcMotor}}$, $W_{13_{R,TcMotor}}$, $W_{14_{L,TcMotor}}$, and $W_{14_{R,TcMotor}}$.

4 SAR CONTROL STRATEGY IMPLEMENTATION

With SAR, all the stride lengths of the right legs will be reduced by reducing the weights of the excitatory signals sent to the Tc motor neurons as follows:

$$W_{k_{L,\ Tc\ Motor}}(t) = \begin{cases} 0.06, & I_1 > 0,\ k = 0, 1, \ldots, 14, \\ 0.3, & I_1 \leq 0,\ k = 0, 1, \ldots, 14, \end{cases} \tag{S14}$$

$$W_{k_{R,\ Tc\ Motor}}(t) = \begin{cases} 0.06, & I_0 > 0,\ k = 0, 1, \ldots, 14, \\ 0.3, & I_0 \leq 0,\ k = 0, 1, \ldots, 14, \end{cases} \tag{S15}$$

where $W_{k_{R,\ Tc\ Motor}}$ is the weight between the right Tc joint and a recurrent neuron on the right Tc series. $W_{k_{L,\ Tc\ Motor}}$ is the weight between the left Tc joint and a recurrent neuron on the left Tc series. To activate the right turn as a result of I_0 activation, all $W_{k_{R,\ Tc\ Motor}}$ will be reduced from 0.3 to 0.06. On the other hand, to activate the left turn as a result of I_1 activation, all $W_{k_{L,\ Tc\ Motor}}$ will be reduced from 0.3 to 0.06. This leads to shorter stride lengths between leg pairs inside the turning curve. These changes in $W_{k_{R,\ Tc\ Motor}}$ or $W_{k_{L,\ Tc\ Motor}}$ of SAR can be distinguished from LCS, where the changes in the stride lengths are adapted based on the changes in the sensory inputs from the SPM instead of the body joint signals from the BBC.

5 GPR CONTROL STRATEGY IMPLEMENTATION

For GPR, the phase of the legs will be reversed according to the sensory signal changes in response to the presence of an obstacle as follows:

$$W_{k_{L,\ Tc\ Motor}}(t) = \begin{cases} -0.3, & I_1 > 0,\ k = 0, 1, \ldots, 14, \\ 0.3, & I_1 \leq 0,\ k = 0, 1, \ldots, 14, \end{cases} \tag{S16}$$

$$W_{k_{R,\ Tc\ Motor}}(t) = \begin{cases} -0.3, & I_0 > 0,\ k = 0, 1, \ldots, 14, \\ 0.3, & I_0 \leq 0,\ k = 0, 1, \ldots, 14, \end{cases} \tag{S17}$$

where $W_{k_{R,\ Tc\ Motor}}$ is the weight between the right Tc joint and a recurrent neuron on the right Tc series. $W_{k_{L,\ Tc\ Motor}}$ is the weight between the left Tc joint and a recurrent neuron on the left Tc series. To activate the right turn as a result of I_0 activation, $W_{k_{R,\ Tc\ Motor}}$ will be reversed from 0.3 to -0.3. On the other hand, to activate the left turn as a result of I_1 activation, $W_{k_{L,\ Tc\ Motor}}$ will be reversed from 0.3 to -0.3. The reversion of $W_{k_{R,\ Tc\ Motor}}$ or $W_{k_{L,\ Tc\ Motor}}$ causes the legs inside the turning arc to move in the opposite direction to the direct-wave gait.

6 SUPPLEMENTARY FIGURE

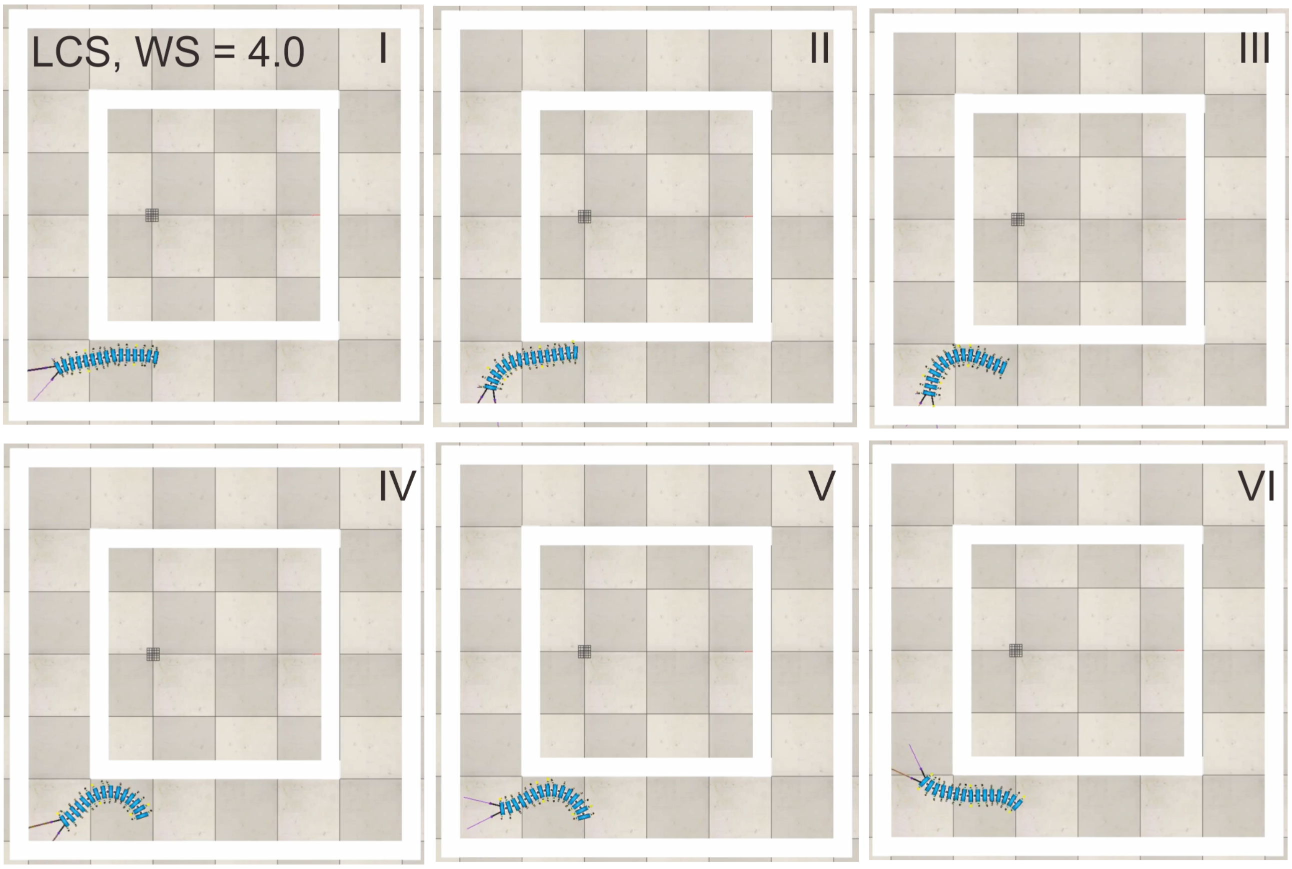

Supplementary Figure S1. Curling behavior of LCS (ws = 4.0). The robot approached a corner in the maze and detected a wall at the corner with its right sensor (state I). It then turned left and persistently detected a wall (state II), leading to a curling behavior (state III). During curling, the left sensor was closer to the wall than the right sensor, resulting in a strong activation to initiate a right turn (state IV). It continued turning right due to the activation of the left sensor (state V) and finally escaped the corner (state VI). Note that this curling behavior could occur at some or all corners and this kind of behaviour was also observed for SAR and GPR at ws = 4.0.
